# Von Willebrand Factor Gene Variants Associate with *Herpes simplex* Encephalitis

**DOI:** 10.1371/journal.pone.0155832

**Published:** 2016-05-25

**Authors:** Nada Abdelmagid, Biborka Bereczky-Veress, Santosh Atanur, Alena Musilová, Václav Zídek, Laura Saba, Andreas Warnecke, Mohsen Khademi, Marie Studahl, Elisabeth Aurelius, Anders Hjalmarsson, Ana Garcia-Diaz, Cécile V. Denis, Tomas Bergström, Birgit Sköldenberg, Ingrid Kockum, Timothy Aitman, Norbert Hübner, Tomas Olsson, Michal Pravenec, Margarita Diez

**Affiliations:** 1 Department of Clinical Neuroscience, Neuroimmunology Unit, Karolinska Institutet, Karolinska University Hospital, SE-171 76 Stockholm, Sweden; 2 Division of Medicine, Imperial College London, London, United Kingdom; 3 Institute of Physiology, Academy of Sciences of the Czech Republic, Prague, Czech Republic; 4 Department of Pharmacology, University of Colorado, Denver School of Medicine, Denver, United States of America; 5 Department of Infectious Diseases, Institute of Biomedicine, Sahlgrenska Academy, University of Gothenburg, Gothenburg, Sweden; 6 Department of Medicine, Unit of Infectious Diseases, Karolinska Institutet, Karolinska University Hospital, SE-171 76 Stockholm, Sweden; 7 Institut National de la Santé et de la Recherche Médicale UMR_S 1176, Univ. Paris-Sud, Université Paris-Saclay, 94276 Le Kremlin-Bicêtre, France; 8 Department of Clinical Virology, University of Gothenburg, SE-413 46 Gothenburg, Sweden; 9 Max-Delbruck-Center for Molecular Medicine, Berlin-Buch, Berlin, Germany; University of Pittsburgh School of Medicine, UNITED STATES

## Abstract

*Herpes simplex* encephalitis (HSE) is a rare complication of *Herpes simplex* virus type-1 infection. It results in severe parenchymal damage in the brain. Although viral latency in neurons is very common in the population, it remains unclear why certain individuals develop HSE. Here we explore potential host genetic variants predisposing to HSE. In order to investigate this we used a rat HSE model comparing the HSE susceptible SHR (Spontaneously Hypertensive Rats) with the asymptomatic infection of BN (Brown Norway). Notably, both strains have HSV-1 spread to the CNS at four days after infection. A genome wide linkage analysis of 29 infected HXB/BXH RILs (recombinant inbred lines—generated from the prior two strains), displayed variable susceptibility to HSE enabling the definition of a significant QTL (quantitative trait locus) named *Hse6* towards the end of chromosome 4 (160.89–174Mb) containing the *Vwf* (von Willebrand factor) gene. This was the only gene in the QTL with both *cis*-regulation in the brain and included several non-synonymous SNPs (single nucleotide polymorphism). Intriguingly, in human chromosome 12 several SNPs within the intronic region between exon 43 and 44 of the VWF gene were associated with human HSE pathogenesis. In particular, rs917859 is nominally associated with an odds ratio of 1.5 (95% CI 1.11–2.02; p-value = 0.008) after genotyping in 115 HSE cases and 428 controls. Although there are possibly several genetic and environmental factors involved in development of HSE, our study identifies variants of the VWF gene as candidates for susceptibility in experimental and human HSE.

## Introduction

HSV-1 (*Herpes simplex* virus type-1) infects the majority of the population resulting in transient cold sores or asymptomatic infection which persists lifelong in the sensory ganglia of the infected individuals. Recurrent herpetic disease occurs after reactivation of HSV-1 from latency in sensory neurons and axonal transport to the periphery. Even though HSV-1 is a widely spread neurotropic virus, herpes simplex encephalitis (HSE) occurs in only 2–3 individuals/million/year and in all ages [1]. More than ninety percent of HSE cases are caused by HSV type-1 and the rest by HSV type-2 [2]. The virus may reach the fronto-temporal lobe via the olfactory tract during primary infection or, more commonly via the trigeminal ganglion after reactivation, resulting in acute aggressive focal necrotizing encephalitis. Patients classically present with fever, headache, altered consciousness, confusion, personality changes, seizures, temporal lobe haemorrhaging and/or other symptoms of focal neurological damage [3, 4]. The disease has a tendency to relapse or have a progressive course [5]. Despite acyclovir treatment, the mortality remains high (6–15%) and among the survivors a high risk of persisting neurological and cognitive deficits remains [6].

We have previously established a rat model of HSE by injecting HSV-1 unilaterally into the whiskers area of inbred DA (Dark Agouti) rats [7]. The *in vivo* model resembles in several aspects the viral spread in human disease, starting from the whiskers area of the rats, corresponding to the labio-facial area in humans. The virus penetrates the peripheral nerve fascicles, spreads then to the trigeminal ganglion, subsequently to the ipsilateral side of the brain stem and then spreads contra-laterally and anteriorly. From 2 dpi (days post-infection) HSV-1 replicates in the perineural cell layer surrounding nerve bundles in the whiskers area [8]. In a model of resistance to HSE, we found that inbred PVG (Piebald Virol Glaxo) rats did not develop HSE because the virus fails to penetrate into the trigeminal nerve. Thus, in PVG rats the CNS (central nervous system) remains uninfected and protected from immunological consequences [7]. We have previously identified the calcitonin receptor gene (*Calcr*) as a candidate for viral spread regulation into the CNS in a F_2_ (DAxPVG.A) intercross [9]. However, in this study we use different rat strains, HSE susceptible SHR (Spontaneously Hypertensive Rats) and asymptomatic infected BN (Brown Norway), that both have viral spread to the nervous system to explore the genetic factors behind HSE development, regardless of the presence of virus in the CNS.

The factors rendering certain hosts; rodents or humans, susceptible to HSE remain unclear. We hypothesized that several variants within the host genome may play a role. Thus, the aims of the present study were to investigate and map any genetic contribution in a rat HSE model and further study the relevance of the findings to human HSE. We identified a genomic region on rat chromosome 4 (*Hse6*) regulating susceptibility *vs*. resistance to HSE using linkage analysis, gene expression in the HXB/BXH RILs (recombinant inbred lines—generated from the prior two strains) [10] and genome sequence analyses in parental strains. The *Vwf* gene was identified as the main candidate gene regulating rat HSE in this set of strain combinations. Moreover, in a human case-control material we identified a nominal association of VWF gene variants.

## Materials and Methods

### Ethics statement

All animal experiments in this study were performed in accordance with the guidelines from the Swedish National Board for Laboratory Animals and the European Community Council Directive (86/609/EEC) and approved by the Swedish ethical committee (Stockholm’s North Ethical Committee—Stockholms Norra Djurförsöksetiska Nämnd) (ethical permit N340/08). Additionally, all human studies enrolment followed the recommendations of the Declaration of Helsinki and the Ethics Committee of the Karolinska Institutet approved the study. Oral and written information was given to the patients and confirmed consent in writing was received before inclusion. (Ethical permit numbers 2002–548, 2004/1-4:6, 2006/1:7, 2008/2:9, 2009/2107-31/2 and 2012/756-31/1).

### Animals

Rats of two inbred strains: the normotensive Brown Norway (BN.*Lx*) and the Spontaneously Hypertensive Rats (SHR/Ola) were used and 29 RILs (recombinant inbred lines; HXB and BXH). The HXB/BXH sets of RILs (n = 29) were derived from F_2_ rats obtained by reciprocal crossings of the Spontaneously Hypertensive Rats (SHR/Ola) and the Brown Norway (BN.*Lx*/Cub) strains [10] followed by brother x sister inbreeding for over 60 generations. The two inbred rats strains and RILs were bred in Prague (Dr. Michal Pravenec’s laboratory) and imported to Karolinska Institutet one week before the experiments were performed. In this article we refer to the parental strains as BN and SHR for convenience.

We used male rats of parental strains BN, SHR, as well as of 19 HXB and 10 BXH RILs. All rats were 45 days old when infected with HSV-1. After infection the rats were observed and followed twice a day for phenotype development until humane endpoint/death or latest until 11 dpi (experimental end point). We defined susceptibility as all rats of a strain reaching a point of no return before dying or dying from HSE and resistance as all rats of a strain being symptom-free and surviving. To detect preclinical signs of disease all rats were weighed daily and examined to detect possible symptoms of HSE development. Animal suffering was minimized by adding extra enrichments, diet pellets on the bedding. However, no pain relievers could be used, since they would have influenced the progression of inflammation. Nevertheless, due to the quick development of the first clinical symptoms to the humane endpoint, the animal suffering in this model was restricted to less than a day. Humane endpoint was defined as a fully developed encephalitis with a point of no return, i.e. when the rats were lying on their side without being able to stand up or crawl. Animals were euthanized using an overdose of barbiturate.

Additional strains were used for haplotype mapping, *i*.*e*. Fisher 344 rats (F344: 5 females, 5 males) generously provided by Professor Holger Luthman, Lund University (Sweden); Lewis rats (LEW: 3 females, 7 males); and spontaneously type 1 diabetic Bio Breeding rats (BB: 1 female, 1 male) from in house breeding [9].

During the experiment rats were kept in a full-barrier animal facility at Portalen Nord, the laboratory animal import station of Karolinska Institutet in groups of 3 to 5 animals per cage under specific pathogen-free and climate-controlled conditions, with 12 hours light/dark cycles. The rats were housed in Eurotype IV cages, in stainless steel isolators (Decco Steel, Sweden) containing aspen wood chips, shavings and chew blocks (Tapvei, Finland) and fed standard rodent chow (SDS, England) and water *ad libitum*. Ambient temperature was 21°C.

### Virus

HSV-1 virus strain I-2762 used was isolated from a brain biopsy taken from a male patient on day 2 after onset of the first clinical symptoms of HSE as described previously [7, 8]. After being thawed to room temperature, 100μl virus suspensions, containing 2 x 10^6^ PFU HSV-1 were injected subcutaneously (*s*.*c*.) into the area of the whiskers’ base unilaterally, on the right side, under 2% Isoflurane (Baxter) anesthesia.

### Immunohistochemistry

Five rats of each parental strain BN and SHR were used for immunohistochemistry and minimum three sections per rat were evaluated from each tissue; whiskers area, trigeminal ganglia and brain stem. The method used for tissue preparation, staining and antibodies were described previously [8]. The antibodies used include polyclonal rabbit anti-HSV-1 (1:100) (Dako), mouse monoclonal anti-Tuj1 (1:500) (Covance), mouse monoclonal anti-ED1 (1:200) (Serotec), mouse monoclonal anti-NKRp1 (1:200) (Harlan Sea-Lab) and mouse monoclonal anti-CD8 (1:200) (Serotec). Additional antibodies used in this study include; polyclonal rabbit anti-von Willebrand factor Ab (1:200) (Abcam) to visualize vWF in the endothelial cell layer and mouse monoclonal anti-Occludin 1A8 Ab (1:200) (Antibodies Online) to visualize the expression of tight junction proteins. After incubation with primary antibodies, sections were rinsed and incubated with Alexa Fluor^™®^488 goat anti-rabbit (1:200) (Molecular Probes, USA) or Alexa Fluor^™®^ 594 goat anti-mouse (1:200) (Molecular Probes) secondary antibodies. Micrographs were taken on a Zeiss Axioskop microscope system and processed in Adobe Photoshop CS3. The pictures included in the Figs represent the staining differences visualised in all slides from the different compartments of all the rats studied.

### QTL Linkage analysis of RILs

QTL linkage analysis correlates the genotypes of each RIL at discrete chromosomal markers with a quantitative phenotype, in this case, incidence of HSE, onset of symptoms, survival days and median weight loss between d0–10 dpi. If there is a strong association between the differences in phenotype and a certain genotype, a QTL will be detected [11]. For the QTL linkage analysis presented in the results section we used median values for all studied phenotypes; these values are summarized in (Fig 3). However, due to some variation in the phenotypes measured for some RILs we also run the linkage analysis including these lines as an intermediate phenotype and they all gave the same QTLs (data not shown, Fig 3). Additionally, we also run the analysis using calculated mean values for all measured phenotypes and these also gave the exact same QTLs (data not shown).

We report loci with genome-wide significance and those considered suggestive are based on 1000 permutation tests that randomly reassign [permute] trait values across the lines and give the thresholds for QTL significance. Whole genome linkage maps were generated using conventional interval mapping allowing linkage analysis across the genome even at points where the genotype data were sparse. Trait values were compared with the probability that a specific genotype exists at a specified location. The LRS (likelihood ratio statistic), a *chi*-square statistic, provides a measure of the linkage between variation in the phenotype and genetic differences at a specific genetic locus, used to identify genome-wide significant QTLs. LOD (logarithm of odds) values can be obtained by dividing the LRS values by 4.6. All linkage maps were generated using WebQTL (The GeneNetwork: http://www.genenetwork.org) and bootstrap analysis was used to evaluate the approximate confidence intervals of QTL peaks.

### Bioinformatic and eQTL analysis

Genome-wide expression data of 15,923 transcripts was previously collected from fat and kidney of 30 RI strains and the SHR and BN progenitor [12] and over 30,000 transcripts from adrenal, kidney, aorta, left ventricle, liver and skeletal muscle [13]. In addition, brain expression data was collected for 198,796 genome-wide transcript clusters (both known/annotated transcripts and computationally predicted transcripts) using the Affymetrix Rat Exon 1.0 ST array. We defined a cis-acting eQTL as a linkage peak for transcript expression within 10 Mbp of the physical location of the transcript. The *cis*-eQTLs in the *Hse6* (chromosome 4: 160.89 Mb to 174 Mb) were extracted using eQTL explorer [14, 15] and the PhenoGen website (http://phenogen.ucdenver.edu; [16]). All eQTLs were calculated with the HXB/BXH panel of recombinant inbred rats using an Affymetrix microarray platform. A small proportion of the eQTLs were detected independently in more than one tissue. These eQTLs can be considered replicated linkages and may reflect common regulatory mechanisms that are shared between tissues.

The genomic SNP variants identified in the SHR and BN strain within the *Hse6* region were extracted using the database SHR base (http://shr.csc.mrc.ac.uk/index.cgi) [17]. To evaluate an eventual functional effect of the variants in the BN genome with respect to their location in annotated genes (ENSEMBL64), ENSEMBL perl APIs database was used. The primer sequences used for *Vwf* SNPs validation in rat using capillary sequencing included the three primers: (Chr.4 position: 161,784,105) forward primer CCTGATGGTCACTGATGGTGAT and reverse primer ACACCGGCGTGTATCAGATG; (position: 161,741,472) forward primer TTGTGTCTGTCCCTCTGACCAT and reverse primer AGTGTGTACCAACCTGCTGCAT; (position: 161,724,332) forward primer TTATTGTCATGAGTCTGGGGTCA and reverse primer CAGGACTCACCGAGAATTGAGA. The SNP haplotype map was generated in RGD (rat genome database).

### Western Blot

Frozen tissue samples from 4 naïve BN and SHR rats were suspended in RIPA buffer (Sigma, R0278) supplemented with Complete^®^ protease inhibitors (Roche). The samples were solubilized by shaking with metal beads (10 min, 50/s) and subsequently sonicated (10 min, 30s on/off). The lysates were cleared by centrifugation (13000 rpm, 4°C, 15 min) to retrieve the protein-containing supernatants. Protein concentrations were determined using the BCA assay (Pierce).

Samples (40μg) were boiled in reducing Laemmli dye (95°C, 5 min) and separated by SDS-PAGE 4–20% MiniPROTEAN^®^ TGX^™^ precast gels (BioRad). After wet transfer to HyBond-ECL nitrocellulose membranes (GE-healthcare), the membranes were blocked using Odyssey^®^ PBS-based Blocking Buffer (LiCOR) and split at the 70kDa marker. The upper part was incubated with polyclonal rabbit anti-vWF (H-300) (Santa Cruz, sc-14014), while the lower part was incubated with polyclonal rabbit anti-Actin (Sigma, A2066) (o.n. 4°C). Membranes were washed 3x with 0.05% PBS-Tween20 and incubated with donkey anti-rabbit IRDye^®^ 800CW (LiCOR) (1h, RT). Unbound antibody was removed by washing 3x with 0.05% PBS-Tween20 and 2x with PBS. Membranes were scanned and quantified using the Odyssey^®^ Clx system (LiCOR).

### Human HSE and controls cohorts

The HSE patients were recruited nation-wide through the national registries using the unique personal identity number following approval of ethical review board. The initial virological diagnosis of these HSE cases was confirmed by the detection of intrathecal HSV-1 antibody production and PCR detection of HSV-1 DNA in CSF samples [18, 19].

The control material we used in this study consisted of a cohort used in part of another nation-wide epidemiological study conducted at Karolinska Institutet focusing on genetic and life-style risk factors for MS. The Epidemiology in MS (EIMS) study consists of incident cases of MS [20]. The inclusion criteria were age between 16 and 70 years of age, recent diagnosis of MS (within two years) and ability to understand Swedish. EIMS controls were selected from the population registry and were matched for sex, age and region of residency. Two controls were identified for each EIMS case. All EIMS cases and controls included in the analysis were sero-positive for HSV-1 IgG as determined by ELISA at the virological laboratory, Sahlgren’s University hospital in Gothenburg, using a whole virus HSV-1 antigen and a confirmative HSV-1 type-specific test using IgG-1 as antigen.

The demographics of the Scandinavian HSE samples included (females n = 64 and males n = 51; age range in years = 15–79 (9 cases <15 years); 4 non-Scandinavian HSE cases (female n = 1 and males n = 3); Scandinavian EIMS cases (females n = 162, males n = 55; age rage in years = 17–68); non-Scandinavian EIMS cases (females n = 23 and males n = 6); Scandinavian EIMS controls (females n = 158, males n = 53; age rage in years = 18–70) and non-Scandinavian controls: (females n = 43 and males n = 8).

### DNA extraction from Human HSE samples

DNA was extracted from whole blood (10–12ml) of 119 HSE cases using standard procedures (QIAamp DNA blood maxi kit, Qiagen). DNA concentrations were measured using an ND-1000 Spectrophotometer (NanoDrop Technologies Inc., Wilmington, DE, USA).

### SNP markers selection and Taqman genotyping

34 SNP markers within the VWF gene were selected for genotyping by running the tagging algorithm, implemented in Haploview 4.2 based on data from the HapMap project using *r*^2^ = 0.8 and LOD 3.0. (http://www.hapmap.org/, release R2/version3/Analysis panel CEU+TSI). We required the tagging SNPs to be present on the Illumina Human 660-quad chip as we used controls typed on this chip. One additional SNP (rs1860545) mapping in a nearby gene which map in an established MS risk gene TNFRSF1A was also added.

All Scandinavian 115 HSE (4 non-Scandinavian HSE) samples and 158 EIMS (epidemiological investigation of multiple sclerosis) project controls were genotyped using Taqman SNP assays from Applied Biosystems (Foster City, CA, USA), as described previously [21]. Assays for seven of the markers (rs10849376, rs216335, rs216340, rs216298, rs2283333, rs1800378 and rs7955850) did not generate consistent genotypes or had poor success rate (<95%) and were excluded from the analysis. Genotypes from Illumina Human660-Quad chip [22] were available for the same markers for the 217 Scandinavian (total = 246) MS cases and 211 Scandinavian (total = 262) population based controls from the EIMS study [20]. All 158 EIMS controls that were genotyped with the Taqman assay had also been genotyped in the Illumina chip. The concordance between the genotyping methods was very high. We followed up the association to rs917859 by genotyping an additionally 6 SNPs (rs12369177, rs7301070, rs2238110, rs917858, rs2239138, rs12829271) in the 119 HSE and 158 EIMS samples. These markers had not been genotyped on the Illumina Human660-Quad chip.

### Statistical analysis for human populations

Association of genotypes in the Scandinavian 115 HSE cases, EIMS 217 MS cases and 211 controls who were positive for HSV-1 IgG was tested using the assoc and hapassoc commands in PLINK v1.06 (http://pngu.mgh.harvard.edu/~purcell/plink/index.shtml). P-values have not been corrected for multiple comparisons. The same analysis was performed including all individuals (Scandinavian and non-Scandinavians) 119 HSE cases and EIMS 246 MS cases and controls. In this study we present data from both cohorts.

## Results

### HSV-1 infects the CNS of the HSE-resistant BN rats

The SHR rats developed HSE after infection with 2 x 10^6^ PFU (plaque-forming unit) HSV-1 and died by 6 dpi. In contrast, the BN rats were clinically resistant, presenting a one day minor weight drop around 5 dpi and thereafter continuing to gain weight corresponding to the weight development of control rats. All BN rats survived until the end point of the experiment designated at 11 dpi and did not show any clinical symptoms of disease. Nonetheless, when previously followed for longer time period, no disease symptoms were detected in the BN rats and they were categorized as clinically resistant.

Paraformaldehyde perfused tissue samples were taken at 4 dpi, before the onset of any symptoms from infected BN and SHR progenitors and subjected to indirect immunohistochemistry staining to visualize the presence of HSV-1, the activation of phagocytic cells (ED1), NK-cells (NKR) and cytotoxic T-cells (CD8) in relation to the nerves in the whiskers area, trigeminal ganglia and in the brain stem. The most striking finding was that HSV-1 penetrated into the trigeminal ganglia and the brain stem of both strains.

**Fig 1:**
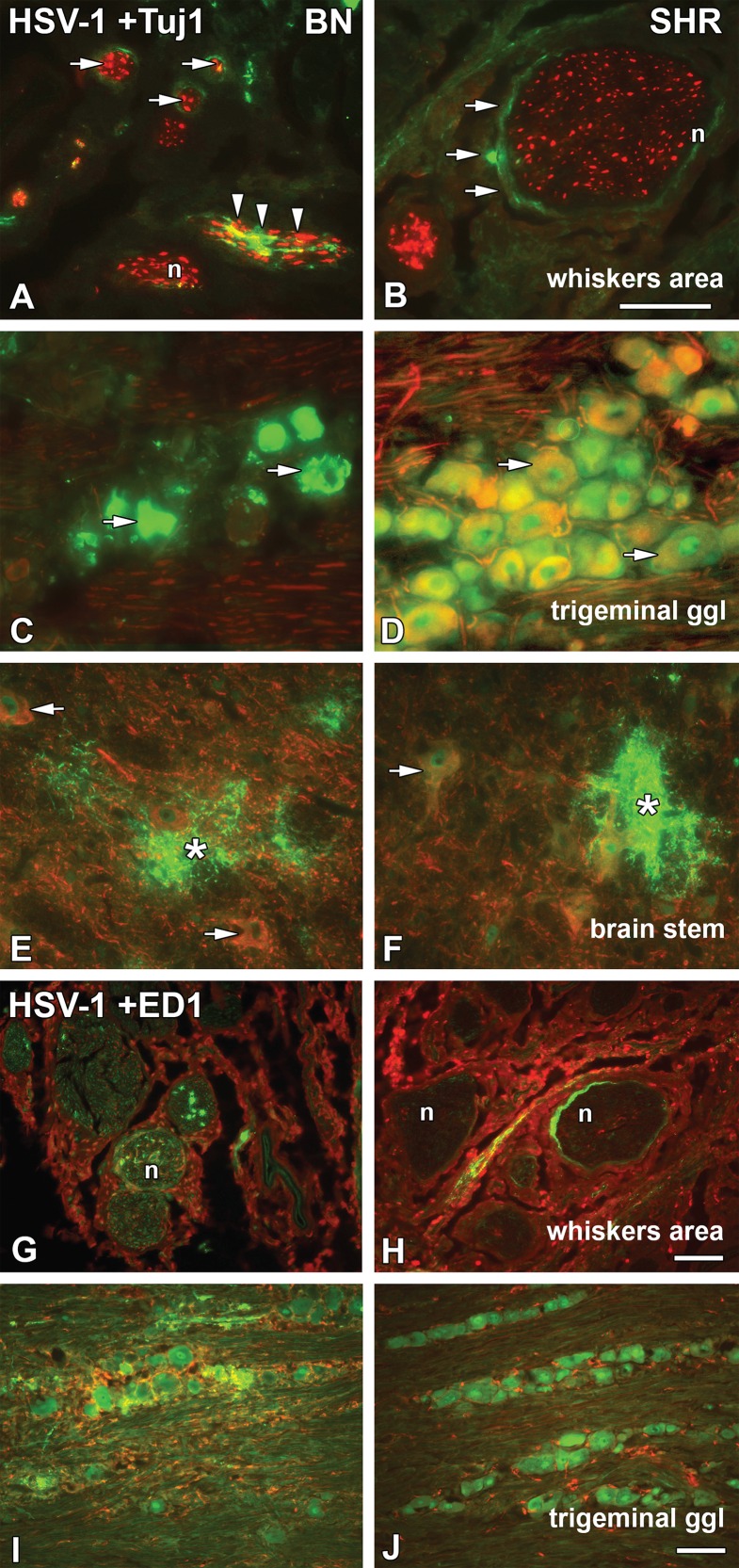
HSV-1 spread in the whiskers area, trigeminal ganglia, brain stem and ED1^+^ phagocytic cells activation. Transversal/coronal sections from the whiskers area (A, B, G and H) and brain stem (E–F), longitudinal/sagittal sections from the trigeminal ganglia (C, D, I and J) from HSV-1 infected BN and SHR strains at 4dpi; stained for HSV-1 marker (green) (A–J), neuronal marker Tuj1 (red) (A–F) and the phagocytic cell marker ED1 (red) (G–J). In the whiskers area, HSV-1 staining was seen in the epineurium of BN (A) and SHR (B) rats, in the perineurial cell layer surrounding nerve fascicles (A and B; arrows) and in the endoneurium of BN rats (A; arrowheads), while sporadically in SHR rats. In the trigeminal ganglia, HSV-1 spread was seen in both strains and was found in the neuronal cell bodies of BN (C; arrows) and SHR (D) rats. However, in the BN rats the staining showed more affected neurons. In the brainstem, HSV-1 staining could be seen mostly in processes surrounding the neurons (E; asterisk) and SHR (F; asterisk). Activation of ED1^+^ phagocytic cells, in the whiskers area could be observed in BN (G) and SHR (H) rats. Phagocytic cells were seen in close proximity to the nerves of the BN rats; whereas in the SHR stronger activation was observed in the outer part of the epineurium (H) and less in the vicinity of the nerve. In the trigeminal ganglia, activated ED1^+^ cells surrounding HSV-1 infected neuronal cell bodies were more visible in BN (I) compared to SHR (J) rats. n = nerve. Scale bar: 50 μm = (A = B = C = D = E = F); (G = H = I = J).

Interestingly, at 4 dpi in the whiskers area we detected the presence of HSV-1 in the epi-, peri- and endoneurium of both BN (Fig 1A) and SHR (Fig 1B) rats. In BN rats, more virus staining was visible inside the nerve fascicles, *i*.*e*. the endoneurium, the layer of connective tissue surrounding the axons within the fascicle (arrowheads in Fig 1A). In both BN and SHR rats HSV-1 was visualized in the perineurium, *i*.*e*. the layer of connective tissue surrounding the nerve fascicles, in ring-like patterns similarly to those seen previously in the susceptible Dark Agouti (DA) rats [8]. The activation of phagocytic cells in the whiskers area was similar in both BN (Fig 1G) and SHR (Fig 1H) strains, with phagocytic cells penetrating into the nerve fascicles.

**Fig 2:**
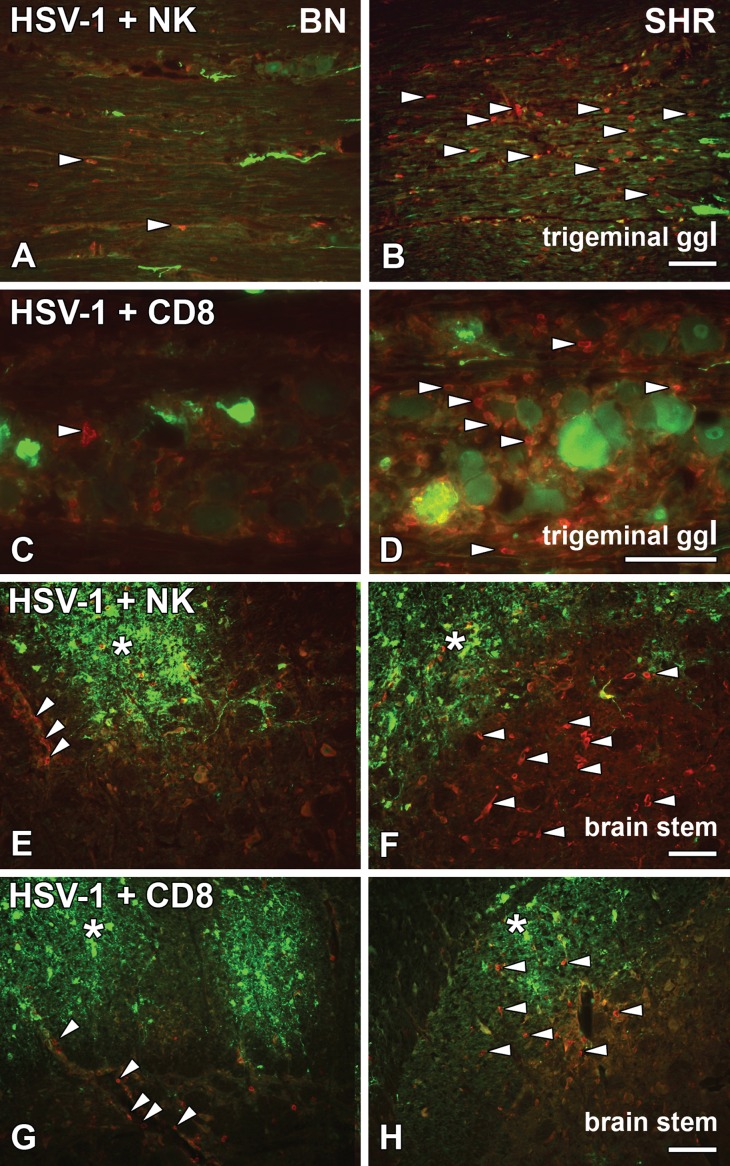
HSV-1 activated NKR^+^ and CD8^+^ cells in the trigeminal ganglia and brain stem. Longitudinal/sagittal sections of the trigeminal ganglia (A–D) and transversal/coronal sections from the brain stem (E–H) from HSV-1 infected BN and SHR rats at 4dpi, stained for HSV-1 marker (green) (A–H), NK cell marker NKR (red) (A, B, E and F) and cytotoxic T cell marker CD8 (red) (C, D, G and H). In the trigeminal ganglia, NK cells were less visible among the axons infected with HSV-1 in the resistant BN rats (A; arrowheads) compared to the SHR rats (B; arrowheads). Also, fewer CD8^+^ cells were observed in BN (C; arrowhead) rats as compared to the SHR (D; arrowheads). While in the brainstem, HSV-1 staining could be seen in both BN (E and G; asterisk) and SHR (F and H; asterisk). NKR^+^ cells were less visible in BN rats (E; arrowheads) outlining the blood vessels as well as cytotoxic CD8^+^ cells (G; arrowheads). In SHR rats, more NK cells (F; arrowheads) and CD8^+^ (H; arrowheads) were present in the parenchyma. Scale bar: 50 μm = (A = B = E = F = G = H), (C = D).

HSV-1 entered the trigeminal ganglia infecting neuronal cell bodies in both the BN (Fig 1C) and SHR (Fig 1D) rats. More virus staining was detected in the neuronal bodies of the BN rats, although they remained asymptomatic. The virus concentration within the cluster of neuronal cells possibly led to the visible disintegration in the neuronal morphology (Figs 1C, 1I and 2C) compared to the neuronal morphology of SHR rats (Figs 1D, 1J and 2D). In the SHR rats HSV-1 infected neurons did not degenerate, nor were ED1+ phagocytic cells in the vicinity of the infected neuronal cell bodies activated in the same extent as in BN rats (Fig 1I and 1J). On the other hand, more NK (Fig 2B) and CD8^+^ cells (Fig 2D) were visible in the trigeminal ganglia of the SHR rats at this time point compared to the BN rats (Fig 2A and 2C).

The viral entry to the CNS was observed in both resistant and susceptible strains at the level of the trigeminal nerve entrance at the ipsilateral side of the brain stem (Figs 1E, 1F and 2E–2H; asterisks). In the brain stem of BN rats, most NKR^+^ cells lined the blood vessels and surrounding tissue (Fig 2E; arrowheads), whereas in the SHR rats NKR^+^ cells were distributed in the brain stem parenchyma (Fig 2F; arrowheads). However, a similar NKR^+^ cells infiltration was observed inside the HSV-1 plaques. The distribution of CD8^+^ cells in the brain stem displayed a similar pattern to NK cells in both BN and SHR (Fig 2G and 2H; arrowheads).

### Genetic analysis in the HXB/BXH RILs

**Fig 3:**
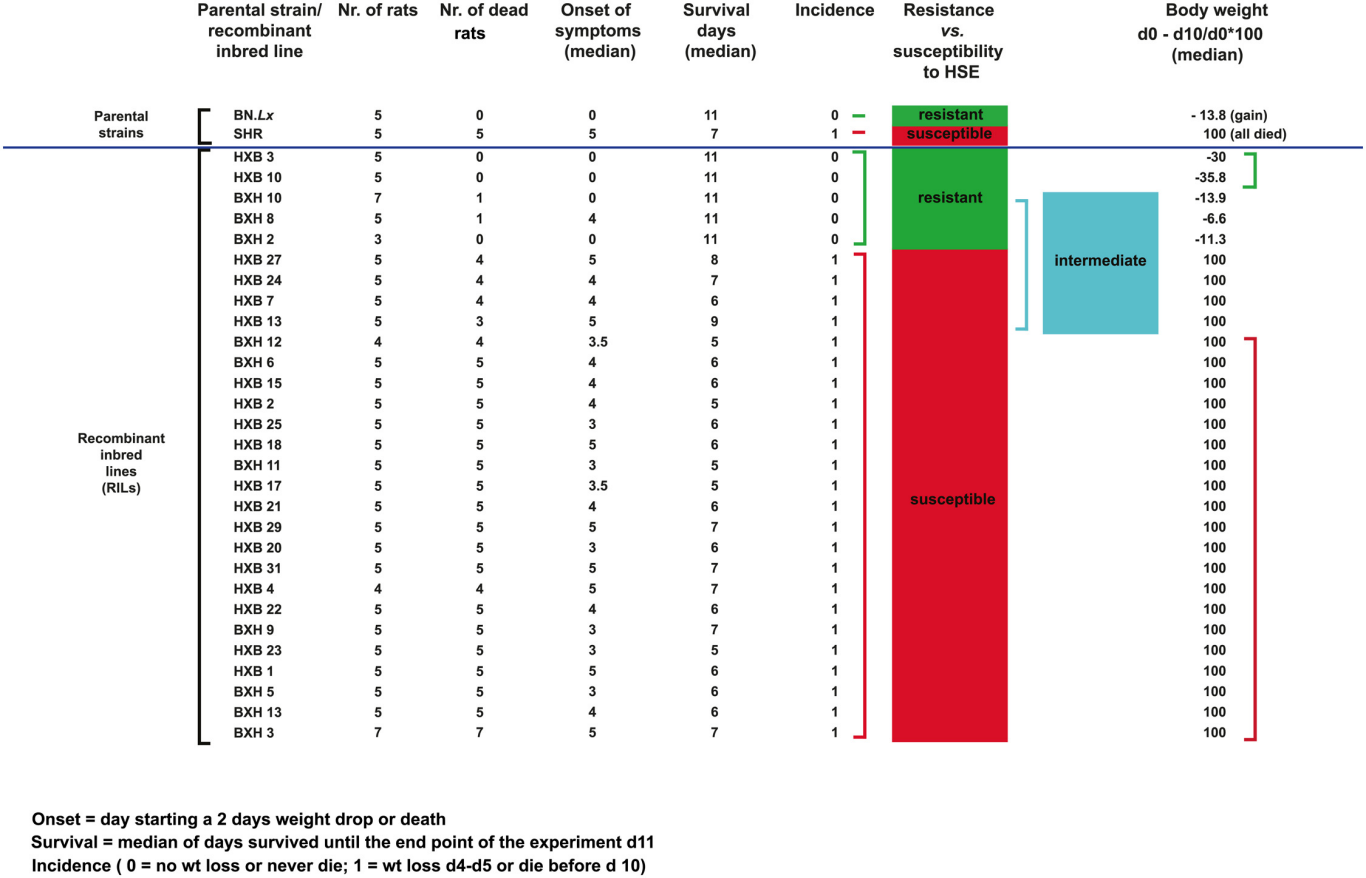
Median values of measured phenotypes after HSV-1 infection in RILs. The table summarizes the median values of measured phenotypes in the parental strains and the 29 RILs 10 days after HSV-1 infection. The table also includes the numbers of rats used from each parental strains and RILs, as well as the number of dead rats from each line.

The genetic determinants of rat HSE were identified using whole genome linkage analysis of the HXB/BXH sets of RILs. The RILs were generated by crossing SHR and BN inbred strains to obtain an F_1_ generation, which were then crossed with each other to generate an F_2_ generation. Breeding pairs from the F_2_ generation were kept separately and bred consecutively by mating siblings for 60 generations. Each of the generated and tested 29 lines had a unique set of recombinations separating them from the other lines [10]. The RILs responded differently to HSV-1 infection mainly in incidence, onset of symptoms and days of survival (Fig 3). Five RILs showed no symptoms of clinical disease and survived until 11 dpi, the experimental end point. Twenty-four RILs presented with disease symptoms between 3 and 5 dpi, while the majority died by 6 dpi (Fig 3). However, when monitoring the weight loss following infection as a sub-phenotype, twenty RILs lost weight, seven RILs had an intermediate phenotype with variations in the degree of weight loss and only two RILs were completely resistant to HSE and did not lose weight at all after HSV-1 infection (Fig 3).

**Fig 4:**
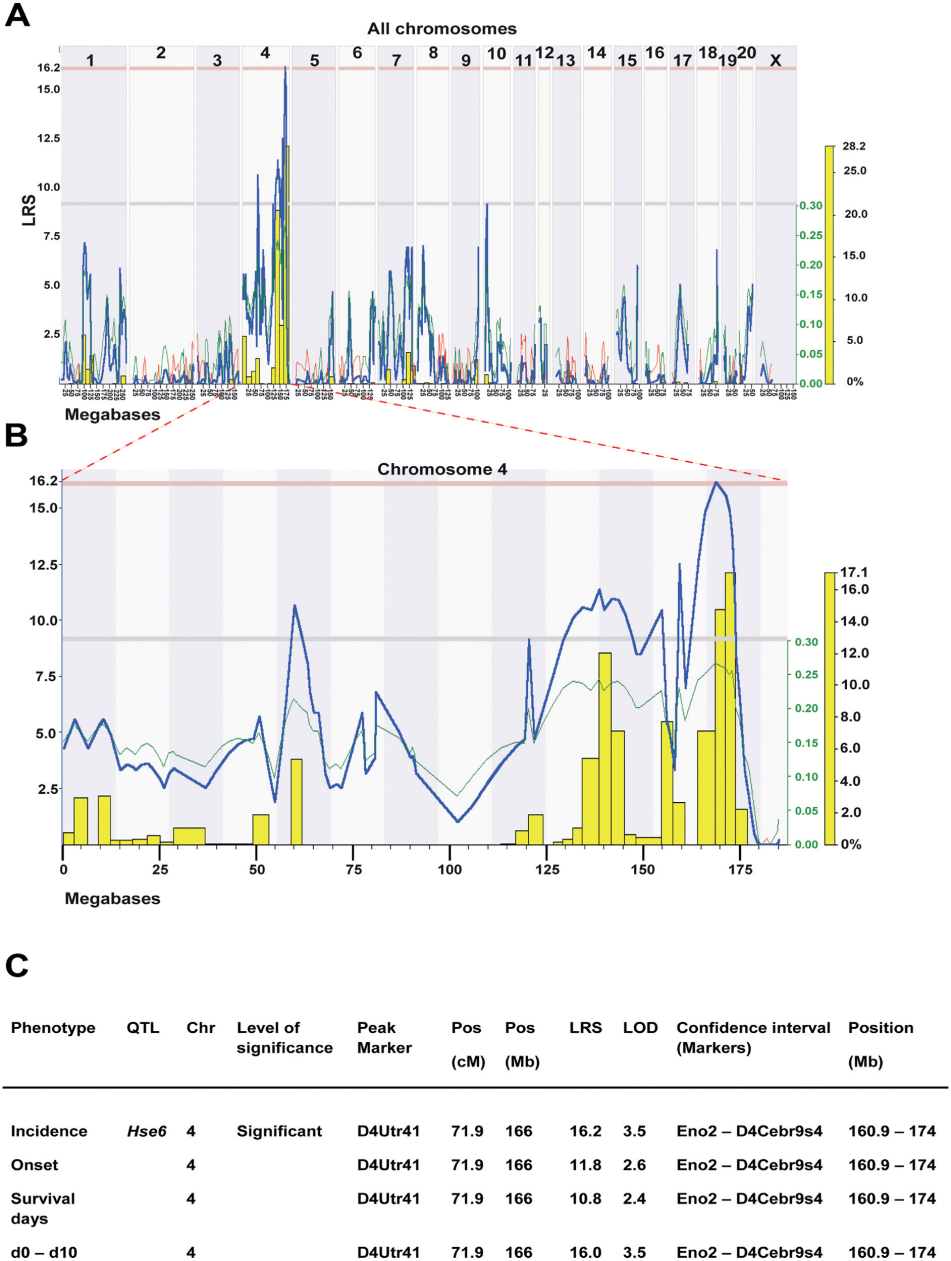
Linkage analysis in RILs reveals a significant QTL on chromosome 4 regulating incidence of HSE. (A) Genome-wide linkage QTL map of HSE incidence. Linkage analysis revealed a significant QTL on chromosome 4 and additional suggestive QTLs on chromosome 4 and 10. The upper x- axis shows rat chromosomes; lower x-axis shows physical map in mega bases and y-axis represents linkage in LRS (likelihood ratio scores) score. Upper red horizontal line: significant LRS genome-wide threshold at *P* ≤ 0.05. Lower grey horizontal line: suggestive LRS genome-wide threshold at *P* ≤ 0.63. WebQTL used to generate the graph. (B) Interval QTL maps with bootstrap analysis of chromosome 4 QTLs for HSE incidence. Yellow-histogram: frequency of peak LRS (bootstrap analysis). The green trace indicates that the SHR allele increase the trait values (positive additive coefficient). WebQTL used to generate the graph. (C) Summary of HSE phenotypes, which are regulated in the *Hse6* QTL in the RILs. Values represent LRS for main effect QTLs. Significance thresholds were generated with 1000 permutations and 1000 bootstraps. The peak marker denoted to the marker closest to the position that showed the maximum LRS for each trait. (To convert LRS to LOD scores, divide by 4.6).

Genome-wide linkage analysis in RILs revealed a significant QTL, *Hse6* regulating HSE incidence on chromosome 4 which also correlated to weight loss (day 0 –day 10) (Fig 4A–4C). This QTL is also suggestive for the regulation of onset of disease and the survival days after infection. The confidence interval (CI) for the *Hse6* is between *Eno2* and D4Cebr9s4 markers (160.89–174 Mb), with D4Utr41 (166 Mb) as the peak marker of QTL linkage. HSE susceptibility is linked to SHR alleles in this region (Fig 4B). A number of additional suggestive QTLs were identified mainly on chromosome 4 for most of the studied phenotypes but also in several other chromosomes. These QTLs include a suggestive QTL on chromosome 1 (CI: 94.3–109.3 Mb) for the survival days, a suggestive QTL on chromosome 9 (CI: 74.7–85.8 Mb) for the difference in weight loss between day 4 and day 5 and a suggestive QTL on chromosome 10 (CI: 12.0–16.7 Mb) for the incidence of disease and survival days. A summary of all the suggestive QTLs that regulate HSE phenotypes detected in HXB/BXH RILs are presented in (S1 Table).

### Searching for candidate genes in the *Hse6* QTL

To search for possible candidate genes in *Hse6* we prioritized significant sequence variants, differentially expressed genes in eight tissues with *cis*-regulation (*i*.*e*. a *cis*-acting expression QTL maps to the physical location of the gene itself [12, 23], and known immune regulatory functions, in the vicinity of the QTL.

The *Hse6* QTL found on chromosome 4 (160.89–174 Mb) associated to incidence of HSE and correlated to weight loss (d0 –d10), harbors about 220 genes. Many of these genes are associated with immune regulation, especially of NK (natural killer) cell and DC (dendritic cell) regulation. This QTL includes a clusters of *Klr* (killer cell lectin-like receptor) genes, which are NK cell receptor genes, *Clec* (C-type lectin family) genes, which are receptors on dendritic and Langerhans cells as well as Ly-49 receptor genes, which are lectin-like (type II transmembrane disulfide-bonded homodimers), expressed on NK cells and some T-cell subsets.

**Fig 5:**
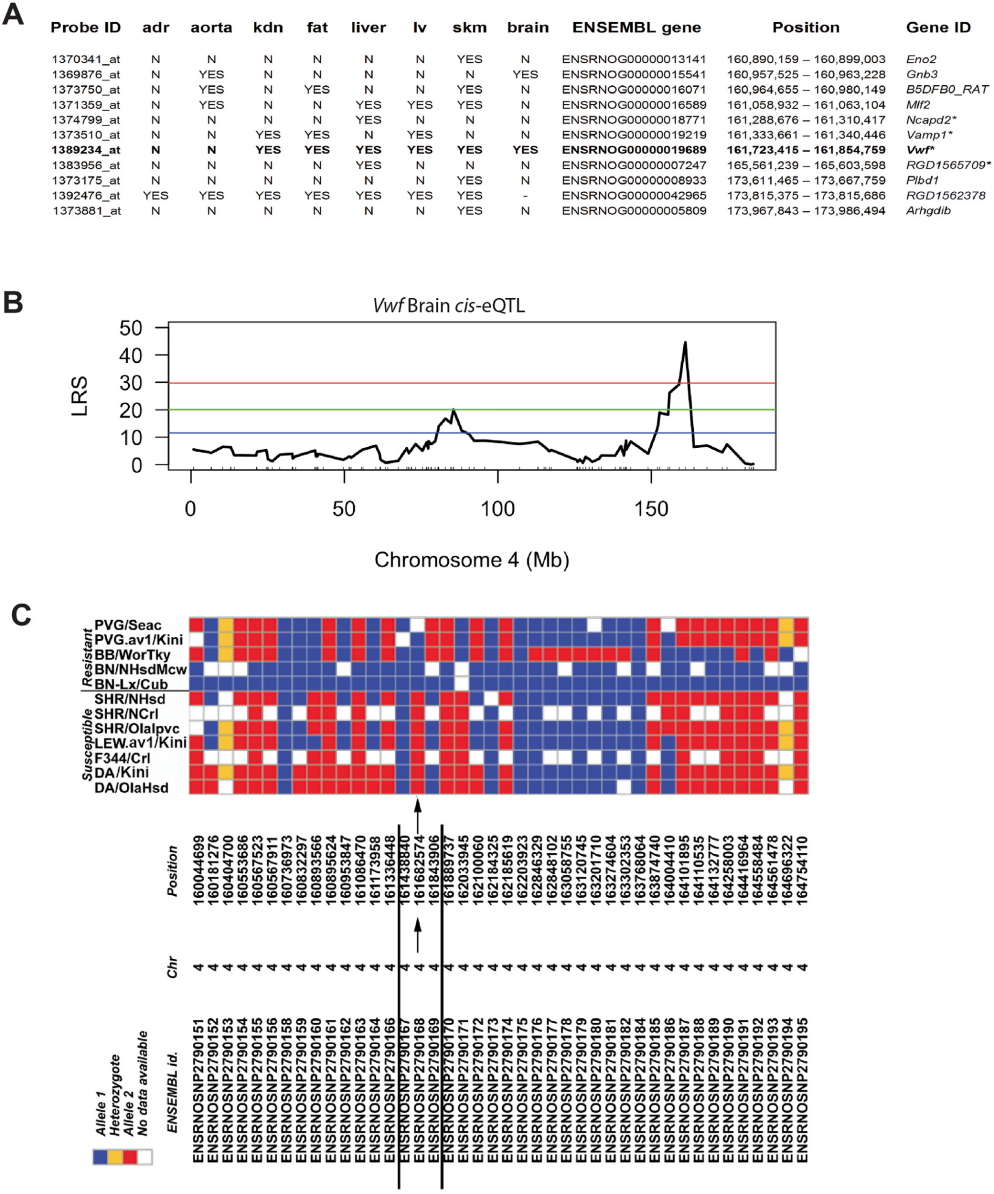
*Vwf* gene is *cis*-regulated in the brain and haplotype mapping revealed a minimal haplotype region. (A) *Cis*-regulated expression QTLs within *Hse6* from naïve tissues of RILs. Abbreviations: adr—adrenal gland; kdn—kidney; lv–left ventricle; skm—skeletal muscle. (B) *Vwf* is significantly *cis*-regulated in the brain eQTL, LRS >40. Upper red horizontal line: significant LRS genome-wide threshold at *P* ≤ 0.05. Lower blue horizontal line: suggestive LRS genome-wide threshold at *P* ≤ 0.63. (C) SNP haplotype map of the tested HSE susceptible and resistant inbred strains and additional not tested sub-colonies of some strains (PVG/seac; BN/NHsdMcw; SHR/NHsd; SHR/Ncrl; DA/Slc; DA/OlaHsd). Within the *Hse6* region, 77 SNPs were found in RGD. Here we present the map with only 42 SNPs (between 160–165 Mb), in which the arrows point towards the only SNP marker position that showed the same allele in all resistant strains (blue allele) and a different allele in all susceptible strains (red allele). The flanking markers define a minimal haplotype region regulating HSE that is spanning over 161,438,840–161,843,906 bp on chromosome 4 indicating the influence of *Vwf gene*.

Comparison of the sequences of the BN and SHR rats in the *Hse6* (160.89–174 Mb) region revealed 6,658 SNPs, 1,861 short indels (insertions/deletions) and 55 large deletions. Out of the 220 genes in this region only 21 genes showed non-synonymous coding variants, out of which only four genes were also *cis*-regulated; *Ncapd2* (ENSEMBL gene ID: ENSRNOG00000018771), *Vamp1* (ENSEMBL gene ID: ENSRNOG00000019219), *Vwf* (ENSEMBL gene ID: ENSRNOG00000019689) and *RGD1565709* (D3ZS19_RAT; ENSEMBL gene ID: ENSRNOG00000007247) (Fig 5A). These 4 genes harbored non-synonymous SNPs and/or variants in putative splice site/promoter region. Strikingly, of all the genes within the QTL, the *Vwf* of SHR origin was the only gene that showed variants in the putative essential splice site (first 2 bps in two introns). *Vwf* also harbored 3 SNP variants upstream of the gene, 2 non-synonymous coding variants; Pro/Leu and Leu/Ser at position 30 and 748 in protein respectively and 18 synonymous variants (S2 Table). To confirm the two non-synonymous variants and the essential splice site variants in *Vwf* gene, we sequenced the region overlapping these variants in both SHR and BN using capillary sequencing by designing primers around ~200 bp on both sides of the variants. All three variants were validated by capillary sequencing. In addition, the *Vwf* gene was *cis*-regulated in 6 out of 8 tissues investigated, including the brain (Fig 5A). The p-values of the brain *Vwf* gene *cis*-eQTLs were 2x10^-4^ and 0.006 respectively using two different probes (Fig 5B) [15].

For detailed exploration of the responsible genetic determinants for susceptibility to HSE a haplotype map was determined in a set of different inbred rat strains. We have previously found that the Lewis (LEW) and Fisher 344 (F344) rats were susceptible to HSE, where all rats from both strains died at 5 dpi, while the Bio Breeding type 1 diabetic rats (BB) were resistant and continued to gain weight after infection [9]. Using rat genome database (RGD) we extracted a SNP haplotype map around *Hse6* region (160–174 Mb; 77 SNPs tested), from different rat strains susceptible and resistant to HSE and additional colonies from some of these strains, to which SNP information was available in RGD. Interestingly, only one marker 4:161682574 showed the same allele in all susceptible strains (DA, LEW, F344, SHR) and another allele in all the resistant ones (PVG, BB, BN). The position of the marker 4:161,682,574 is approximately 40kb upstream to the *Vwf* gene. The flanking markers are spaced 243,734 bp and 161,332 bp on each side and define a minimal haplotype region that is spanning over 161,438,840–161,843,906 bp on chromosome 4 (Fig 5C, arrows). A critical aspect for haplotype analysis is that genes elsewhere in the genome may convey resistance. Therefore, the PVG rat was excluded from this haplotype analysis since it is possibly resistant due to variants in the *Calcr* gene leading to lack of viral entry into the CNS [9]. However, the sharing of the same marker in four susceptible strains and another allele in the resistant BB and BN strains is interesting.

Within this minimal haplotype region only 3 genes were encoded, tumour necrosis factor receptor superfamily member 3 (*Ltbr*, ENSEMBL gene ID: ENSRNOG00000019264), Amiloride-sensitive sodium channel subunit alpha (*Scnn1a*, ENSEMBL gene ID: ENSRNOG00000019368) and *Vwf*. Eight SNP variants were found upstream of the *Ltbr* gene, 14 SNPs were found 340–4200 bp upstream of the *Scnn1a* gene and 3 were found 2677–4561 bp upstream of the *Vwf* gene. One non-synonymous SNP variant located in a putative coding/splice site was found in *Scnn1a* (transcript ENSRNOT00000026320) and in *Vwf*, one non-synonymous coding variant was found in exon 2 and one in exon 17, but none in *Ltbr*. *Vwf* also harbors a SNP variant located at the putative essential splice site for exon 6.

### Von Willebrand factor (vWF) protein expression in BN and SHR tissues

**Fig 6:**
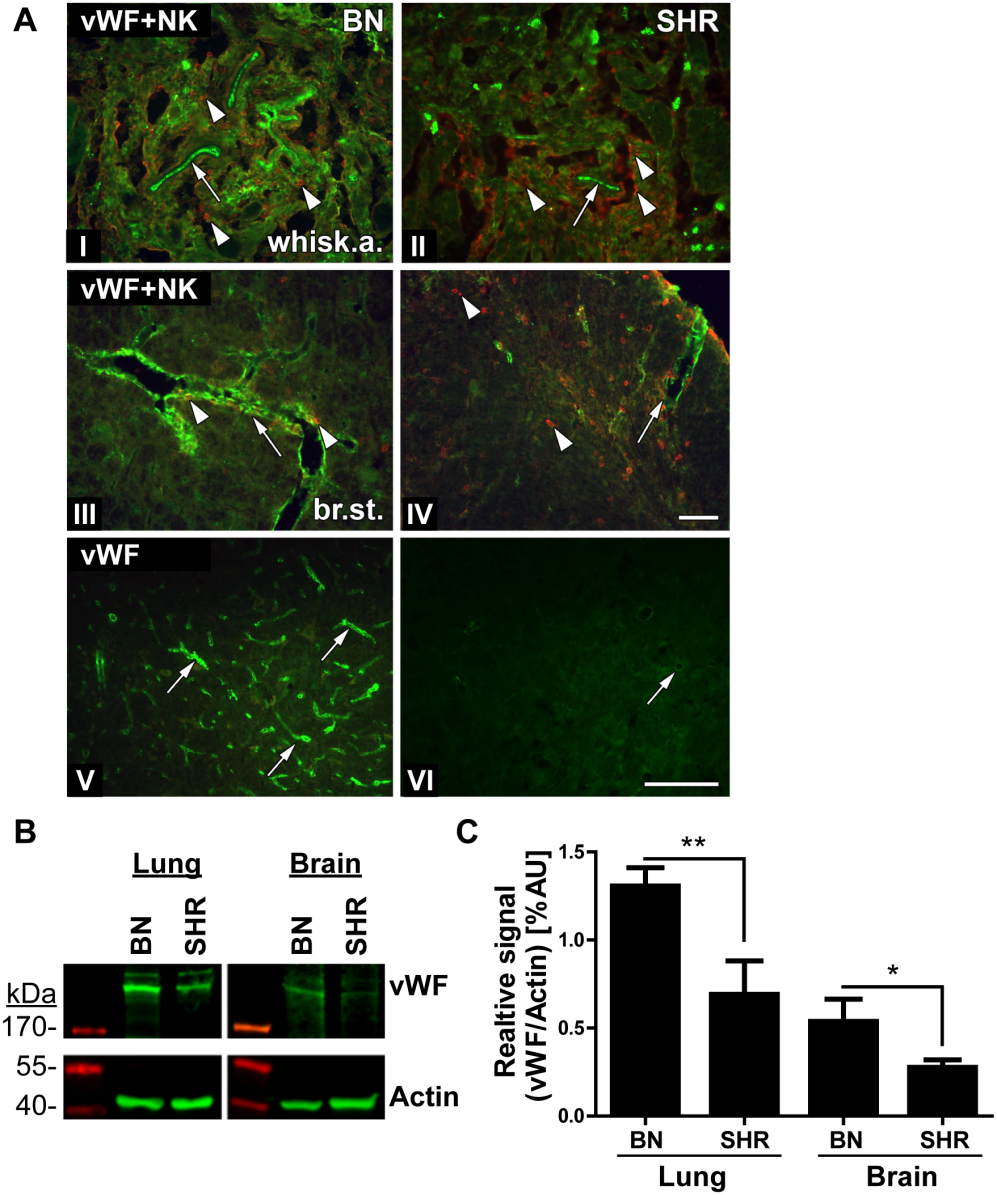
Less expression of vWF in the brain of SHR rats. (A) Transversal/coronal sections from the whiskers area (I and II) and the brain stem (III–VI) of BN (I, III and V) and SHR (II, IV and VI) rats at 4dpi, stained with vWF Ab (green) (I–VI), NK cell marker NKR (red) (I–IV). Distinct staining of vWF could be observed in the whiskers area (I; arrow) and the brain stem (III; arrow) of the BN rats with a limited spread of NK cells (I and III; arrowheads) compared to SHR. In SHR, lower and more fragmented staining of vWF (II and IV; arrows) could be observed with more abundant influx of NK cells both in the whiskers area (B; arrowheads) and brain stem parenchyma (IV; arrowheads). vWF staining was visible in the brain stem of the BN rats outlining capillaries (V; arrows). While in the SHR rats was reduced and fragmented in the larger vessels (IV and VI; arrows) and absent in the small capillaries (VI). Scale bar: 50 μm = (I = II = III = IV); (V = VI). (B) vWF expression assessed by Western Blot: The image displays representative samples from naïve lung and brain tissues from BN and SHR rats. The expression of vWF was more abundant and significantly higher in the lungs as compared to brain tissues in both strains. SHR strain displayed reduced levels of vWF in both tissues compared to BN. Actin served as a loading control. (C) Quantification of vWF expression assessed using Western Blot: We quantified the vWF expression from bands above 200kDa and normalized the signal to the respective actin controls. The data includes quantifications from 3–4 naïve rats/group. In both tissues, BN rats had a significantly higher vWF expression compared to SHR as analysed by Student’s T-test (*: p<0.05, **p<0.01).

Considering all the findings in this study, *Vwf* was the main candidate gene for regulating the difference in susceptibility to HSE between the studied rat strains. Using immunohistochemistry we assessed staining differences in vWF expression in HSV-1 infected BN and SHR rats (Fig 6A). The vWF protein is synthesized in the endothelial cells and is stored in intracellular granules. vWF functions in the blood coagulation system as platelets adhesion molecule and anti-haemophilic factor carrier in vessel walls, having a role in hemostasis [24]. In addition, it has also been suggested to have a role in maintaining the blood-brain barrier hemostasis [25].

In the whiskers area and in the brain stem of BN rats, vWF staining clearly delineated the large blood vessels and capillaries (Fig 6A(I), 6A(III) and 6A(V); arrows). However, in the SHR rat vWF staining was less visible around the blood vessels (Fig 6A(II), 6A(IV) and 6A(VI), arrows). In parallel, we stained the tight junction protein, occludin as a marker for endothelial cells and the BBB (blood brain barrier) to assess its integrity in BN and SHR rats after HSV-1 infection. In the brain stem of both strains, the occludin staining outlined the larger blood vessels with no visible difference between these two strains, indicating that there were no major differences in the staining of the tight junctions in the two strains (S1 Fig).

To quantify the vWF expression in BN and SHR rats we performed Western Blot on naive brain lysates and vessel-rich lungs as controls. In both tissues, the BN rats displayed a significantly higher expression of vWF compared to SHR (Fig 6B and 6C). Thus, the Western Blots support the assessment of higher vWF expression in BN rats observed in histological staining of brain sections.

### rs917859 SNP within the VWF gene is associated with human HSE

To investigate the association of VWF gene to human HSE disease, we genotyped 27 SNPs within the VWF gene and one SNP within the TNFRSF1A adjacent gene located on chromosome 12 using Taqman in 115 HSE cases and 158 EIMS (epidemiological investigation of multiple sclerosis project) controls [20]. In the analysis, we also included 217 EIMS MS cases and 211 EIMS controls that were serum positive to HSV-1 infection. We found a nominal association with the SNP rs917859 (5.95 Mb) with odds ratio (OR) of 1.5 (p-value = 0.008) (Table 1). Interestingly, the association seems to be of a recessive pattern (S3 Table). This SNP is located within the intronic region between exon 43 and 44 of the VWF gene. We tested haplotype association between rs917859 and the adjacent markers (Table 2) in which the rs917859 –rs4764521 GG haplotype was slightly more significantly associated (p-value = 0.004) to HSE than the rs917859 marker alone.

**Table 1:** Summary of VWF SNPs analysis and association in HSE cases and EIMS controls.

						Scandinavian		All individuals	
Marke	Position	Minor allele	MAF cases	MAF controls	HWE P controls	OR (95% CI)	P-value	OR (95% CI)	P-value
rs2885517	5920581	tagged	0.61	0.58		0.79	0.26	1.12	0.44
rs2270151	5931221	A	0.1282	0.1585	0.869	0.77 (0.50–1.19)	0.24	0.78 (0.51–1.19)	0.2461
rs2286646	5931752	G	0.2155	0.2165	0.1159	1.04 (0.73–1.49)	0.81	0.99 (0.70–1.41)	0.9729
rs12317523	5933403	A	0.2564	0.2579	0.7291	0.96 (0.69–1.35)	0.82	0.99 (0.72–1.37)	0.9632
rs723188	5934166	A	0.1838	0.1627	0.03447	1.27 (0.87–1.86)	0.21	1.16 (0.80–1.68)	0.4363
rs12368267	5941777	G	0.09322	0.1063	0.4879	0.91 (0.57–1.49)	0.70	0.86 (0.53–1.40)	0.553
rs11063961	5944277	G	0.1907	0.187	0.0403	1.00 (0.69–1.45)	0.98	1.02 (0.71–1.47)	0.8965
rs12300917	5949319	A	0.1496	0.1903	0.08273	0.80 (0.54–1.21)	0.29	0.75 (0.51–1.11)	0.1458
rs917857	5952085	A	0.4153	0.4783	0.6567	0.75 (0.55–1.09)	**0.05**	0.77 (0.58–1.03)	0.0806
**rs917859**	**5952374**	**A**	0.4231	0.3287	0.3659	1.50 (1.11–2.02)	**0.008**	1.50 (1.12–2.00)	**0.006253**
rs4764521	5954584	A	0.2542	0.2126	0.5962	1.27 (0.91–1.79)	0.16	1.26 (0.91–1.76)	0.1645
rs216883	5967918	A	0.2974	0.2638	0.5678	1.13 (0.82–1.57)	0.45	1.18 (0.86–1.62)	0.2978
rs216889	5969714	A	0.4873	0.4882	0.4787	0.99 (0.74–1.33)	0.92	1.00 (0.75–1.32)	0.9801
rs17491334	5974105	A	0.08547	0.1201	0.6792	0.73 (0.44–1.20)	0.21	0.68 (0.42–1.12)	0.1326
rs216904	5976279	tagged	0.43	0.39		0.73	0.21	1.15	0.33
rs216312	5999245	A	0.4545	0.4803	0.6567	0.93 (0.68–1.25)	0.62	0.90 (0.67–1.21)	0.4877
rs11611917	6006895	A	0.2119	0.252	0.9062	0.86 (0.60–1.23)	0.42	0.80 (0.57–1.12)	0.1965
rs216338	6018587	A	0.3559	0.378	1	0.95 (0.70–1.29)	0.74	0.91 (0.68–1.22)	0.5288
rs980130	6039288	A	0.3008	0.3278	0.1067	0.87 (0.63–1.19)	0.39	0.88 (0.65–1.20)	0.4257
rs980131	6039459	tagged	0.39	0.42		0.84	0.29	0.86	0.33
rs4764482	6039994	G	0.5388	0.4901	0.01605	0.82 (0.61–1.11)	0.20	1.22 (0.91–1.62)	0.1808
rs12319392	6040747	A	0.07203	0.08858	1	0.86 (0.50–1.50)	0.60	0.80 (0.47–1.37)	0.4127
rs2283332	6044990	A	0.09402	0.09055	1	1.00 (0.60–1.67)	0.98	1.04 (0.64–1.70)	0.8681
rs3782711	6053442	G	0.1154	0.1181	0.8313	1.00 (0.64–1.58)	0.99	0.97 (0.62–1.52)	0.9071
rs2238104	6057926	A	0.5042	0.4419	0.02437	1.32 (0.98–1.77)	0.06	1.28 (0.97–1.71)	0.08327
rs2239144	6066444	tagged	0.10	0.13		0.71	0.17	0.80	0.34
rs2239140	6070704	A	0.4872	0.4557	0.0897	1.15 (0.86–1.55)	0.35	1.14 (0.85–1.51)	0.384
rs11064024	6072310	G	0.3771	0.3984	0.3089	0.96 (0.71–1.30)	0.81	0.92 (0.68–1.22)	0.5464
rs11836843	6073079	G	0.06356	0.06496	0.7128	0.98 (0.55–1.78)	0.97	0.98 (0.55–1.74)	0.9372
rs1860545	6317038	A	0.4103	0.4341	0.1765	0.93 (0.69–1.26)	0.65	0.91 (0.68–1.21)	0.5073

MA = minor allele; MAF = minor allele frequency; HWE = Hardy-Weinberg Equilibrium; OR = odds ratio; CI = confidence interval.

Success rate for each marker in cases and controls was between (98–100%). Chr12 SNPs positions (in bp) (Genome build 36.3).

Tagging with two or three markers was used for 4 markers: rs2885517 was tagged by the rs2270151 A—rs12317523 G haplotype;

rs216904 was tagged by the rs17491334 A—rs216889 G haplotype; rs980131 was tagged by the rs12319392 C—rs4764482 A haplotype and rs2239144 was tagged by the rs2238104 C—rs2239140 G -rs11064024 A haplotype.

Scandinavian columns: represent analysis of OR and P-value made in Scandinavian 115 HSE cases and 428 EIMS controls;

All individuals columns: represent analysis of OR and P-value made in Scandinavian and non-Scandinavian 119 HSE cases and 508 EIMS controls.

**Table 2:** Haplotype association between rs917859 and the two adjacent markers.

				Scandinavians		All individuals	
Marker combination	Haplotype	Frequency cases	Frequency controls	OR	P-value	OR	P-value
rs917857—rs917859	GA	0.42	0.33	1.49	0.008	1.51	0.006
	AG	0.41	0.48	0.75	0.05	0.78	0.08
	GG	0.16	0.19	0.87	0.43	0.82	0.24
rs917859—rs4764521	AA	0.12	0.10	1.48	0.21	1.43	0.17
	GA	0.13	0.12	1.15	0.60	1.16	0.53
	AG	0.30	0.23	1.43	0.04	1.45	0.03
	GG	0.45	0.56	0.63	0.004	0.62	0.003

OR = odds ratio

Scandinavian columns: represent analysis of OR and P-value made in Scandinavian 115 HSE cases and 428 EIMS controls;

All individuals columns: represent analysis of OR and P-value made in Scandinavian and non-Scandinavian 119 HSE cases and 508 EIMS controls.

**Fig 7:**
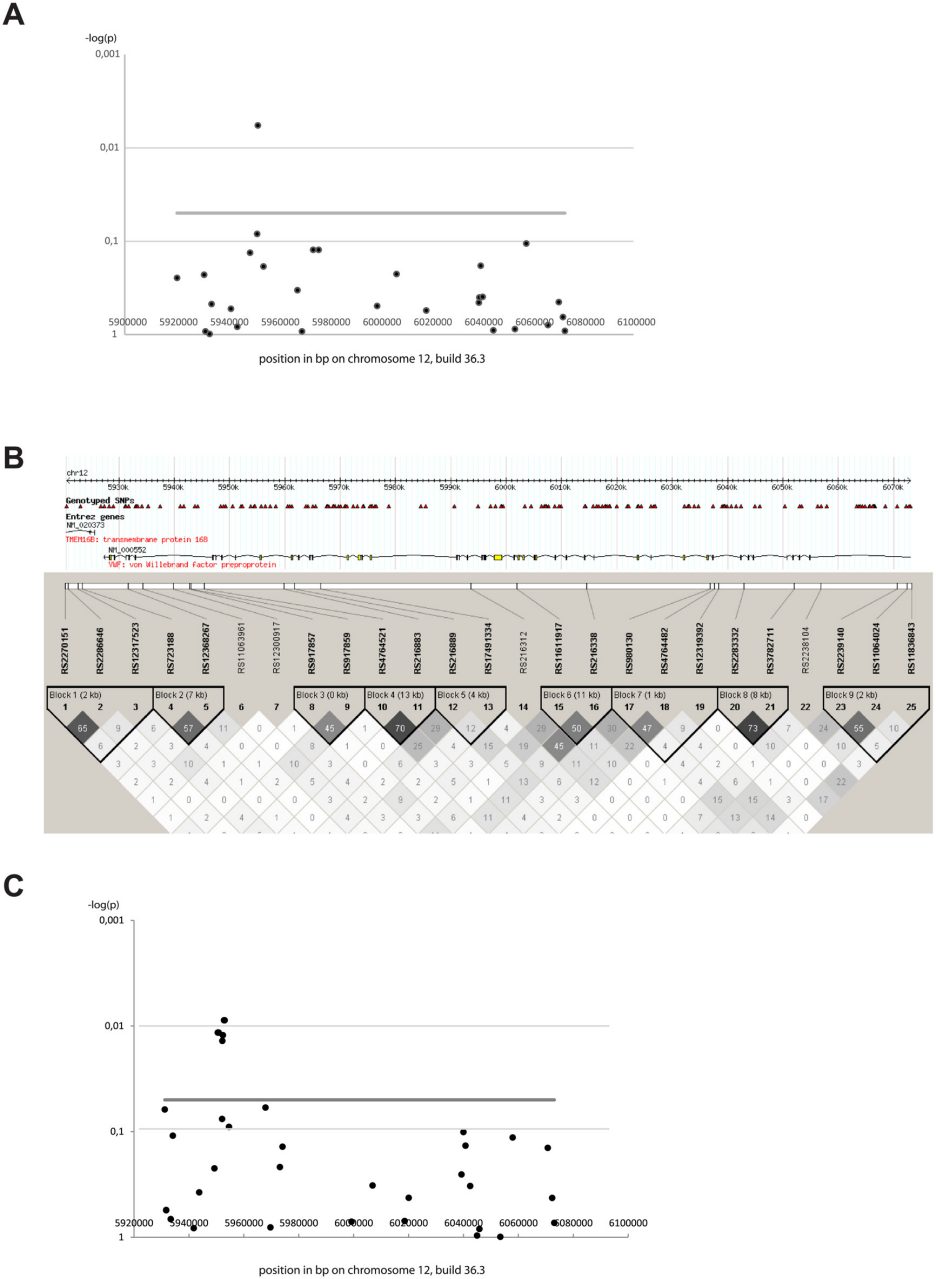
Illustration of VWF SNPs positions, linkage disequilibrium and association to HSE. (A) Association was tested in Scandinavian and non-Scandinavian 119 HSE patients and 508 control individuals positive for HSV-1 IgG antibodies. Evidence of association is plotted as the negative logarithm to the base 10 of the p-value of allelic association against position in base pair along chromosome 12. The association was estimated using the assoc and hap-assoc (for aggressively tagged markers) commands in PLINK. The genomic build used was 36.3. (B) Linkage disequilibrium (LD) measured by r2 between markers included in the association analysis. The LD was estimated in Haploview 4.2 using genotypes from 119 HSE patients and 508 individuals positive for HSV-1 IgG antibodies. (C) Association plot including the additional 6 SNPs tested in 119 HSE patients and 158 EIMS controls positive for HSV-1 IgG antibodies. Evidence of association is plotted as the negative logarithm to the base 10 of the p-value of allelic association against position in base pair along chromosome 12.

Additionally, we genotyped 6 SNPs within the intronic region between exon 43 and 44 that showed association in all HSE cases and the 158 EIMS controls. We identified association to HSE with more SNPs in this region, especially rs2239138 and rs12829271 with OR of 1.6 (p-value = 0.06) (Table 3). Haplotype association for these new markers and rs917859 generated similar p-values as for single markers (data not shown). Also, all the analysis was performed including more HSE cases and controls of non-Scandinavian origins. The association was mapped to the same SNP variants with increased significance (Fig 7, Tables 1–3).

**Table 3:** Summary of additional VWF SNPs analysis and association in 115 HSE cases and 158 EIMS controls.

					Scandinavian		All individuals	
SNP marker	Position	Minor Allele	MAF cases	MAF controls	OR (95% CI)	P-value	OR (95% CI)	P-value
**rs12369177**	5943781	T	0.404	0.366	0.97 (0.65–1.43)	0.86	1.18 (0.81–1.70)	0.39
rs11063961	5944277	G	0.191	0.187	1.00 (0.69–1.45)	0.98	1.02 (0.71–1.47)	0.90
rs12300917	5949319	A	0.149	0.190	0.80 (0.54–1.21)	0.29	0.75 (0.51–1.11)	0.15
**rs7301070**	5950551	G	0.423	0.314	1.46 (0.98–2.16)	0.06	1.61 (1.11–2.33)	0.01
**rs2238110**	5950838	G	0.423	0.314	1.46 (0.98–2.16)	0.06	1.61 (1.11–2.33)	0.01
rs917857	5952085	A	0.415	0.478	0.75 (0.55–1.00)	0.05	0.77 (0.58–1.03)	0.08
**rs917858**	5952199	C	0.423	0.316	1.43 (0.97–2.13)	0.07	1.59 (1.09–2.30)	0.01
rs917859	5952374	A	0.423	0.329	1.50 (1.11–2.02)	0.008	1.50 (1.12–2.00)	0.006
**rs2239138**	5952819	A	0.427	0.314	1.46 (0.98–2.16)	0.06	1.63 (1.13–2.37)	0.009
**rs12829271**	5953060	A	0.427	0.314	1.46 (0.98–2.16)	0.06	1.63 (1.13–2.37)	0.009
rs4764521	5954584	A	0.254	0.213	1.27 (0.91–1.79)	0.16	1.26 (0.91–1.76)	0.16

MAF = minor allele frequency; HWE = Hardy-Weinberg Equilibrium; OR = odds ratio; CI = confidence interval. (Chr12 SNPs positions (in bp) (Genome build 36.3). Table summarizing the analysis of the new 6 and 3 associated SNPs (the analysis was run in a total of 36 SNPs, data not shown).

Scandinavian columns: represent analysis of OR and P-value made in Scandinavian 115 HSE cases and 428 EIMS controls;

All individuals columns: represent analysis of OR and P-value made in Scandinavian and non-Scandinavian 119 HSE cases and 508 EIMS controls.

In conclusion, the association of human VWF SNP variants to HSE in this region, and thus the confirmation of the rat data, further supports the novel association of VWF to HSE.

## Discussion

HSE, although rare, remains the most common cause of acute sporadic viral encephalitis in the western world [26]. Despite the adequate use of antiviral treatment, the mortality and morbidity remains high and therefore dissection of the pathogenic factors is warranted. The key findings of the current study point to genetic variants of the VWF gene, together with expression differences as a novel risk for HSE development. Though first identified in young adult rats as one main candidate factor determining HSE susceptibility, the findings were replicated in a human case-control cohort and several variants in the VWF gene were nominally associated with HSE.

We studied HSE pathogenesis using an experimental animal model. HSV-1 was detected in the whiskers area, trigeminal ganglia and the brain stem in both BN and SHR strains. The presence of the virus in the parenchyma of resistant BN rats oppose our previous findings in the resistant PVG rats, in which HSV-1 did not penetrate the CNS [7]. In mice, a similar phenomenon of infection has been described earlier in both resistant and susceptible strains [27]. In spite of the viral spread to the trigeminal ganglia in both rat strains a more pronounced infection and phagocytic activation was observed in the neurons of the resistant BN rats. However, in the susceptible SHR strain more NK and CD8^+^ cells were visualised indicating a possibly stronger cytotoxic immune reaction at 4 dpi, which subsequently may contribute to the development of disease symptoms in this strain. This is in line with the previously published results indicating a combined role of NK and CD8^+^ cells in regulating virus spread in the HSE resistant C57BL/6 mice [28]. However, the difference in immune responses detected in our studied strains could also be due to variants in immune related genes and not exclusively linked to the identified variants in the *Vwf* gene. Additional studies will be needed to dissect these differences and understand the exact mechanism of disease. It has previously been reported in paediatric case studies that impaired production of type I interferons (IFN) via the toll-like receptor 3 (TLR3) pathway were involved in HSE susceptibility. In these cases the influence was attributed mainly to genetic polymorphisms in UNC93B1 [29]and TLR3 genes [30]. Also, a number of studies using different HSE mice models and/or reverse genetics approaches point to the influence of different immune cells, cytokines and chemokines in viral reactivation and disease outcome [31–34]. Altogether, these findings support the notion of a role of immune genes in disease pathogenesis. Additionally, from our previous findings in rat, we identified *Calcr* as a candidate for HSE susceptibility in DA and PVG strains [9] suggesting the involvement of multiple genes in HSE regulation.

In our study, the use of genome wide linkage analysis unraveled the genomic region *Hse6* regulating HSE. *Hse6* harbors a number of immune-related genes, which might have relevance in the susceptibility to/protection against HSE development such as the NK and dendritic cell receptor genes. An earlier study in mice identified a natural killer (NK) complex-linked locus, *Rhs1* (resistance to *Herpes simplex* virus 1), on mouse chromosome 6 that controls resistance to acute and latent HSV-1 infections [35]. However, our sequencing and expression data combined with the haplotype analysis narrowed down the numerous candidates in *Hse6* to the *Vwf* gene. Additionally, the study by Lundberg and colleagues identified in a mouse model of corneal HSV-1 infection a second locus on chromosome 6, *Hrl* (Herpes resistance locus) to influence survival to HSV-1 infection in C57BL/6J. The relatively large confidence interval of *Hrl* contains many genes, including the *Tnfrsf1a* gene suggested as a candidate for resistance in the mouse species [36], which is located in close proximity to the *Vwf* gene highlighted in our study. Recently, another study using a different model in mice maps between Cd69 and D6Wum34, to a segment separate from the previous mice QTLs that also consists of NK complex-linked locus that confers resistance to HSV-1 infection of the brain [37]. Thus, all the regions described in mice restrict viral spread to the CNS and correspond to the same *Hse6* region in rat. Interestingly, the co-localization of the QTLs both in rats and mice provide an additional support to the influence of this region on the HSE pathogenesis in rodents and the importance of NK cells for anti-viral responses.

The exact role of *Vwf* variants on HSE susceptibility still remains to be understood and further mechanistic studies are needed. The vWF is a large glycoprotein found in blood plasma, platelet α-granules, endothelial cells and subendothelial connective tissue. The vWF protein has an important role in hemostasis, regulating the adhesion of platelets and the binding of factor VIII [24]. In this study, our immunohistochemical stainings indicated less abundant vWF around blood vessels in the susceptible SHR strain, which was supported by quantification of the protein using Western Blot. The reduced expression may be attributed to the SNP variants in the *Vwf* gene. Since the expression of vWF is reduced in the HSE susceptible SHR strain, we speculate a possible dysregulation in the CNS endothelial cell layer. This could lead to an increased vascular permeability or alternative mechanisms in SHR rats leading to more infiltration of immune cells into the CNS after HSV-1 infection. Also, we previously published, using a rat heterogeneous stock (HS) composed of eight different outbred rat strains, that *Vwf* was the main gene in a locus regulating platelet aggregation. This further indicates that the presence of variations within the *Vwf* gene in different rat strains is likely to contribute in influencing additional phenotypes [38]. It has been reported previously when using *Vwf* knockout mice in an experimental autoimmune/allergic encephalomyelitis (EAE) model for Multiple Sclerosis (MS), that vWF influences the permeability of the blood brain barrier (BBB) leading to increased infiltration of inflammatory cells into the CNS [39]. The interaction between different viruses and BBB cells causing increased permeability and cellular infiltration has been proposed in a number of studies, even though the exact mechanisms are not yet well defined. This has be shown after infections with West Nile virus (WNV) [40, 41], Japanese encephalitis virus (JEV) [42], rabies virus [43] and HIV [44]. In line with these findings, HSV-1 infection in the CNS could possibly cause a disruption of the endothelial BBB lining entailing increased cellular infiltration through VWF dependent or independent mechanisms. Notably, in the plasma of cerebral malaria patients the release of increased levels of vWF has been reported after *Plasmodium falciparum* infection [45–48]. Together, this highlights a role of vWF after infection with different pathogens particularly in the CNS.

We believe that our finding of a nominal association of the VWF gene to human HSE disease is of clinical interest and may be translated to predictive markers. Interestingly, the data also suggest a recessive pattern of inheritance. A replication of the association in a larger and independent cohort would be desirable and of value for future studies. Furthermore, mechanistic studies of vWF and the interplay with immune-related genes that govern HSE susceptibility are required.

## Conclusions

Our work identified *Vwf* as a candidate for HSE regulation in rat. Although HSV-1 was spread to the CNS of both rat strains used, only the susceptible SHR strain showed more NK cell infiltration. Notably, the expression of vWF was reduced in the susceptible strain. We also investigated an involvement of VWF in human HSE by the nominal association of several SNP variants. Collectively, these data implicate the importance of controlling cell activation and infiltration during neuroinflammation and neuropathogenesis of HSE. The mechanisms underlying HSE need to be further explored in relation to allelic variants of VWF and other risk genes discovered in rodents models and humans.

## Supporting Information

S1 Fig Similar occludin staining in the blood vessels of the brain stem of both strains. Transversal/coronal sections from the brain stem of BN (A, B, C, G, H and I) and SHR (D, E, F, J, K and L) rats stained with HSV-1 Ab (green) (A, C, D and F), occludin (tight junctions marker) (red) (B, C, E, F, H, I, K and L) and vWF marker (green) (G and J). The HSV-1 spread was seen in the brain stem of the resistant BN rats (A and C; asterisks) and the susceptible SHR (D and F; asterisks). vWF protein was found in the large vessels and capillaries of BN rats in the brain stem (G and I; arrowheads and arrow) while in the SHR rats vWF staining was only visible in the larger vessels (J and L; arrowheads). Occludin staining of the tight junctions was similar in the brain stem of both BN (B, C, H and I; arrows) and SHR (E, F, K and L; arrows) rats. Scale bar: 50 μm.
(TIF)

S1 Table Summary of identified suggestive QTLs that regulate HSE phenotypes in RILs. (DOC)

S2 Table List of SNPs within the *cis*-regulated genes in *Hse6*. (DOC)

S3 Table Summary of VWF SNPs analysis and association in HSE cases and EIMS controls using recessive test. (DOCX)

## Abbreviations

HSE: *Herpes simplex* virus type-1 encephalitis
BN: Brown Norway rat
SHR: Spontaneously hypertensive rat
RILs: recombinant inbred lines
QTL: quantitative trait locus
*Vwf* or VWF: Von Willebrand factor gene
vWF: Von Willebrand factor protein

## References

[1] Hjalmarsson A, Granath F, Forsgren M, Brytting M, Blomqvist P, Skoldenberg B. Prognostic value of intrathecal antibody production and DNA viral load in cerebrospinal fluid of patients with herpes simplex encephalitis. J Neurol. 2009;256(8):1243–51. Epub 2009/04/09. 10.1007/s00415-009-5106-6 . 19353228

[2] Aurelius E, Johansson B, Skoldenberg B, Forsgren M. Encephalitis in immunocompetent patients due to herpes simplex virus type 1 or 2 as determined by type-specific polymerase chain reaction and antibody assays of cerebrospinal fluid. J Med Virol. 1993;39(3):179–86. Epub 1993/03/01. . 8385702 10.1002/jmv.1890390302

[3] Kennedy PG, Chaudhuri A. Herpes simplex encephalitis. J Neurol Neurosurg Psychiatry. 2002;73(3):237–8. Epub 2002/08/20. 12185148 10.1136/jnnp.73.3.237 PMC1738005

[4] Whitley RJ. Viral encephalitis. The New England journal of medicine. 1990;323(4):242–50. Epub 1990/07/26. 10.1056/NEJM199007263230406 . 2195341

[5] Sköldenberg B, Aurelius E, Hjalmarsson A, Sabri F, Forsgren M, Andersson B, et al Incidence and pathogenesis of clinical relapse after herpes simplex encephalitis in adults. J Neurol. 2006;253(2):163–70. . 16222428 10.1007/s00415-005-0941-6

[6] Studahl M, Lindquist L, Eriksson BM, Gunther G, Bengner M, Franzen-Rohl E, et al Acute viral infections of the central nervous system in immunocompetent adults: diagnosis and management. Drugs. 2013;73(2):131–58. Epub 2013/02/05. 10.1007/s40265-013-0007-5 . 23377760

[7] Bereczky-Veress B, Lidman O, Sabri F, Bednar I, Granath F, Bergstrom T, et al Host strain-dependent difference in susceptibility in a rat model of herpes simplex type 1 encephalitis. J Neurovirol. 2008;14(2):102–18. Epub 2008/04/30. 792702050 [pii] 10.1080/13550280701883832 . 18444082

[8] Bereczky-Veress B, Abdelmagid N, Piehl F, Bergstrom T, Olsson T, Skoldenberg B, et al Influence of Perineurial Cells and Toll-Like Receptors 2 and 9 on Herpes simplex Type 1 Entry to the Central Nervous System in Rat Encephalitis. PLoS One. 2010;5(8). Epub 2010/09/02. e12350 [pii] 10.1371/journal.pone.0012350 20806060 PMC2929186

[9] Abdelmagid N, Bereczky-Veress B, Guerreiro-Cacais AO, Bergman P, Luhr KM, Bergstrom T, et al The Calcitonin Receptor Gene Is a Candidate for Regulation of Susceptibility to Herpes simplex Type 1 Neuronal Infection Leading to Encephalitis in Rat. PLoS pathogens. 2012;8(6):e1002753 Epub 2012/07/05. 10.1371/journal.ppat.1002753 22761571 PMC3386237

[10] Pravenec M, Klir P, Kren V, Zicha J, Kunes J. An analysis of spontaneous hypertension in spontaneously hypertensive rats by means of new recombinant inbred strains. J Hypertens. 1989;7(3):217–21. Epub 1989/03/01. . 2708818

[11] Williams RW, Gu J, Qi S, Lu L. The genetic structure of recombinant inbred mice: high-resolution consensus maps for complex trait analysis. Genome Biol. 2001;2(11):RESEARCH0046. Epub 2001/12/12. 11737945 10.1186/gb-2001-2-11-research0046 PMC59991

[12] Hubner N, Wallace CA, Zimdahl H, Petretto E, Schulz H, Maciver F, et al Integrated transcriptional profiling and linkage analysis for identification of genes underlying disease. Nat Genet. 2005;37(3):243–53. Epub 2005/02/16. ng1522 [pii] 10.1038/ng1522 . 15711544

[13] Heinig M, Petretto E, Wallace C, Bottolo L, Rotival M, Lu H, et al A trans-acting locus regulates an anti-viral expression network and type 1 diabetes risk. Nature. 2010;467(7314):460–4. Epub 2010/09/10. 10.1038/nature09386 . 20827270 PMC3657719

[14] Mueller M, Goel A, Thimma M, Dickens NJ, Aitman TJ, Mangion J. eQTL Explorer: integrated mining of combined genetic linkage and expression experiments. Bioinformatics. 2006;22(4):509–11. Epub 2005/12/17. btk007 [pii] 10.1093/bioinformatics/btk007 . 16357031

[15] Tabakoff B, Saba L, Printz M, Flodman P, Hodgkinson C, Goldman D, et al Genetical genomic determinants of alcohol consumption in rats and humans. BMC Biol. 2009;7:70 Epub 2009/10/31. 1741-7007-7-70 [pii] 10.1186/1741-7007-7-70 19874574 PMC2777866

[16] Bhave SV, Hornbaker C, Phang TL, Saba L, Lapadat R, Kechris K, et al The PhenoGen informatics website: tools for analyses of complex traits. BMC Genet. 2007;8:59 Epub 2007/09/01. 10.1186/1471-2156-8-59 17760997 PMC2034588

[17] Atanur SS, Birol I, Guryev V, Hirst M, Hummel O, Morrissey C, et al The genome sequence of the spontaneously hypertensive rat: Analysis and functional significance. Genome Res. 2010 Epub 2010/05/01. gr.103499.109 [pii] 10.1101/gr.103499.109 . 20430781 PMC2877576

[18] Sköldenberg B, Forsgren M, Alestig K, Bergström T, Burman L, Dahlqvist E, et al Acyclovir versus vidarabine in herpes simplex encephalitis. Randomised multicentre study in consecutive Swedish patients. Lancet. 1984;2(8405):707–11. . 6148470 10.1016/s0140-6736(84)92623-0

[19] Aurelius E, Johansson B, Skoldenberg B, Staland A, Forsgren M. Rapid diagnosis of herpes simplex encephalitis by nested polymerase chain reaction assay of cerebrospinal fluid. Lancet. 1991;337(8735):189–92. Epub 1991/01/26. . 1670839 10.1016/0140-6736(91)92155-u

[20] Hedstrom AK, Baarnhielm M, Olsson T, Alfredsson L. Tobacco smoking, but not Swedish snuff use, increases the risk of multiple sclerosis. Neurology. 2009;73(9):696–701. Epub 2009/09/02. 10.1212/WNL.0b013e3181b59c40 . 19720976

[21] Ekelund E, Saaf A, Tengvall-Linder M, Melen E, Link J, Barker J, et al Elevated expression and genetic association links the SOCS3 gene to atopic dermatitis. Am J Hum Genet. 2006;78(6):1060–5. Epub 2006/05/11. 10.1086/504272 16685656 PMC1474106

[22] Sawcer S, Hellenthal G, Pirinen M, Spencer CC, Patsopoulos NA, Moutsianas L, et al Genetic risk and a primary role for cell-mediated immune mechanisms in multiple sclerosis. Nature. 2011;476(7359):214–9. Epub 2011/08/13. 10.1038/nature10251 21833088 PMC3182531

[23] Petretto E, Mangion J, Dickens NJ, Cook SA, Kumaran MK, Lu H, et al Heritability and tissue specificity of expression quantitative trait loci. PLoS Genet. 2006;2(10):e172 Epub 2006/10/24. 06-PLGE-RA-0248R2 [pii] 10.1371/journal.pgen.0020172 17054398 PMC1617131

[24] Sadler JE. Biochemistry and genetics of von Willebrand factor. Annu Rev Biochem. 1998;67:395–424. Epub 1998/10/06. 10.1146/annurev.biochem.67.1.395 . 9759493

[25] Suidan GL, Brill A, De Meyer SF, Voorhees JR, Cifuni SM, Cabral JE, et al Endothelial Von Willebrand factor promotes blood-brain barrier flexibility and provides protection from hypoxia and seizures in mice. Arterioscler Thromb Vasc Biol. 2013;33(9):2112–20. 10.1161/ATVBAHA.113.301362 23825365 PMC3854012

[26] Sancho-Shimizu V, Zhang SY, Abel L, Tardieu M, Rozenberg F, Jouanguy E, et al Genetic susceptibility to herpes simplex virus 1 encephalitis in mice and humans. Curr Opin Allergy Clin Immunol. 2007;7(6):495–505. Epub 2007/11/09. 17989525 10.1097/ACI.0b013e3282f151d2

[27] Halford WP, Balliet JW, Gebhardt BM. Re-evaluating natural resistance to herpes simplex virus type 1. J Virol. 2004;78(18):10086–95. . 15331741 10.1128/JVI.78.18.10086-10095.2004 PMC515006

[28] Kastrukoff LF, Lau AS, Takei F, Smyth MJ, Jones CM, Clarke SR, et al Redundancy in the immune system restricts the spread of HSV-1 in the central nervous system (CNS) of C57BL/6 mice. Virology. 2010 Epub 2010/03/05. S0042-6822(10)00113-3 [pii] 10.1016/j.virol.2010.02.013 . 20199790

[29] Casrouge A, Zhang SY, Eidenschenk C, Jouanguy E, Puel A, Yang K, et al Herpes Simplex Virus Encephalitis in Human UNC-93B Deficiency. Science. 2006;314:308–12. . 16973841 10.1126/science.1128346

[30] Zhang SY, Jouanguy E, Ugolini S, Smahi A, Elain G, Romero P, et al TLR3 deficiency in patients with herpes simplex encephalitis. Science. 2007;317(5844):1522–7. . 17872438 10.1126/science.1139522

[31] Pasieka TJ, Cilloniz C, Carter VS, Rosato P, Katze MG, Leib DA. Functional genomics reveals an essential and specific role for Stat1 in protection of the central nervous system following herpes simplex virus corneal infection. J Virol. 2011;85(24):12972–81. 10.1128/JVI.06032-11 21994441 PMC3233176

[32] Wickham S, Lu B, Ash J, Carr DJ. Chemokine receptor deficiency is associated with increased chemokine expression in the peripheral and central nervous systems and increased resistance to herpetic encephalitis. J Neuroimmunol. 2005;162(1–2):51–9. Epub 2005/04/19. 10.1016/j.jneuroim.2005.01.001 . 15833359

[33] Zhou Y, Lu ZN, Guo YJ, Mei YW. Favorable effects of MMP-9 knockdown in murine herpes simplex encephalitis using small interfering RNA. Neurol Res. 2010;32(8):801–9. 10.1179/016164110X12644252260556 . 20483026

[34] Sheridan BS, Cherpes TL, Urban J, Kalinski P, Hendricks RL. Reevaluating the CD8 T-cell response to herpes simplex virus type 1: involvement of CD8 T cells reactive to subdominant epitopes. J Virol. 2009;83(5):2237–45. 10.1128/JVI.01699-08 19073721 PMC2643732

[35] Pereira RA, Scalzo A, Simmons A. Cutting edge: a NK complex-linked locus governs acute versus latent herpes simplex virus infection of neurons. J Immunol. 2001;166(10):5869–73. Epub 2001/05/09. . 11342599 10.4049/jimmunol.166.10.5869

[36] Lundberg P, Welander P, Openshaw H, Nalbandian C, Edwards C, Moldawer L, et al A locus on mouse chromosome 6 that determines resistance to herpes simplex virus also influences reactivation, while an unlinked locus augments resistance of female mice. J Virol. 2003;77(21):11661–73. Epub 2003/10/15. 14557652 10.1128/JVI.77.21.11661-11673.2003 PMC229335

[37] Kastrukoff LF, Lau AS, Takei F, Carbone FR, Scalzo AA. A NK complex-linked locus restricts the spread of herpes simplex virus type 1 in the brains of C57BL/6 mice. Immunol Cell Biol. 2015 Epub 2015/05/15. 10.1038/icb.2015.54 . 25971711

[38] Rat Genome S, Mapping C, Baud A, Hermsen R, Guryev V, Stridh P, et al Combined sequence-based and genetic mapping analysis of complex traits in outbred rats. Nat Genet. 2013;45(7):767–75.; PubMed Central PMCID: PMC3821058. 23708188 10.1038/ng.2644 PMC3821058

[39] Noubade R, del Rio R, McElvany B, Zachary JF, Millward JM, Wagner DD, et al von-Willebrand factor influences blood brain barrier permeability and brain inflammation in experimental allergic encephalomyelitis. Am J Pathol. 2008;173(3):892–900. Epub 2008/08/09. ajpath.2008.080001 [pii] 10.2353/ajpath.2008.080001 18688020 PMC2526288

[40] Roe K, Orillo B, Verma S. West Nile virus-induced cell adhesion molecules on human brain microvascular endothelial cells regulate leukocyte adhesion and modulate permeability of the in vitro blood-brain barrier model. PLoS One. 2014;9(7):e102598 10.1371/journal.pone.0102598 25036379 PMC4103843

[41] Daniels BP, Holman DW, Cruz-Orengo L, Jujjavarapu H, Durrant DM, Klein RS. Viral pathogen-associated molecular patterns regulate blood-brain barrier integrity via competing innate cytokine signals. MBio. 2014;5(5):e01476–14. 10.1128/mBio.01476-14 25161189 PMC4173776

[42] Li F, Wang Y, Yu L, Cao S, Wang K, Yuan J, et al Viral Infection of the Central Nervous System and Neuroinflammation Precede Blood-Brain Barrier Disruption during Japanese Encephalitis Virus Infection. J Virol. 2015;89(10):5602–14. 10.1128/JVI.00143-15 25762733 PMC4442524

[43] Chai Q, He WQ, Zhou M, Lu H, Fu ZF. Enhancement of blood-brain barrier permeability and reduction of tight junction protein expression are modulated by chemokines/cytokines induced by rabies virus infection. J Virol. 2014;88(9):4698–710. 10.1128/JVI.03149-13 24522913 PMC3993813

[44] Davidson DC, Hirschman MP, Sun A, Singh MV, Kasischke K, Maggirwar SB. Excess soluble CD40L contributes to blood brain barrier permeability in vivo: implications for HIV-associated neurocognitive disorders. PLoS One. 2012;7(12):e51793 10.1371/journal.pone.0051793 23251626 PMC3520914

[45] Hollestelle MJ, Donkor C, Mantey EA, Chakravorty SJ, Craig A, Akoto AO, et al von Willebrand factor propeptide in malaria: evidence of acute endothelial cell activation. Br J Haematol. 2006;133(5):562–9. Epub 2006/05/10. 10.1111/j.1365-2141.2006.06067.x . 16681646

[46] Bridges DJ, Bunn J, van Mourik JA, Grau G, Preston RJ, Molyneux M, et al Rapid activation of endothelial cells enables Plasmodium falciparum adhesion to platelet-decorated von Willebrand factor strings. Blood. 2010;115(7):1472–4. Epub 2009/11/10. 10.1182/blood-2009-07-235150 19897581 PMC2840836

[47] de Mast Q, Groot E, Lenting PJ, de Groot PG, McCall M, Sauerwein RW, et al Thrombocytopenia and release of activated von Willebrand Factor during early Plasmodium falciparum malaria. J Infect Dis. 2007;196(4):622–8. Epub 2007/07/13. 10.1086/519844 . 17624850

[48] Larkin D, de Laat B, Jenkins PV, Bunn J, Craig AG, Terraube V, et al Severe Plasmodium falciparum malaria is associated with circulating ultra-large von Willebrand multimers and ADAMTS13 inhibition. PLoS pathogens. 2009;5(3):e1000349 Epub 2009/03/21. 10.1371/journal.ppat.1000349 19300493 PMC2652105

