# Supplementary figures and images for: Von Willebrand Factor Gene Variants Associate with Herpes simplex Encephalitis
Source: PLoS One. 2016 May 25;11(5):e0155832. doi: 10.1371/journal.pone.0155832 (PMC4880288; doi:10.1371/journal.pone.0155832)

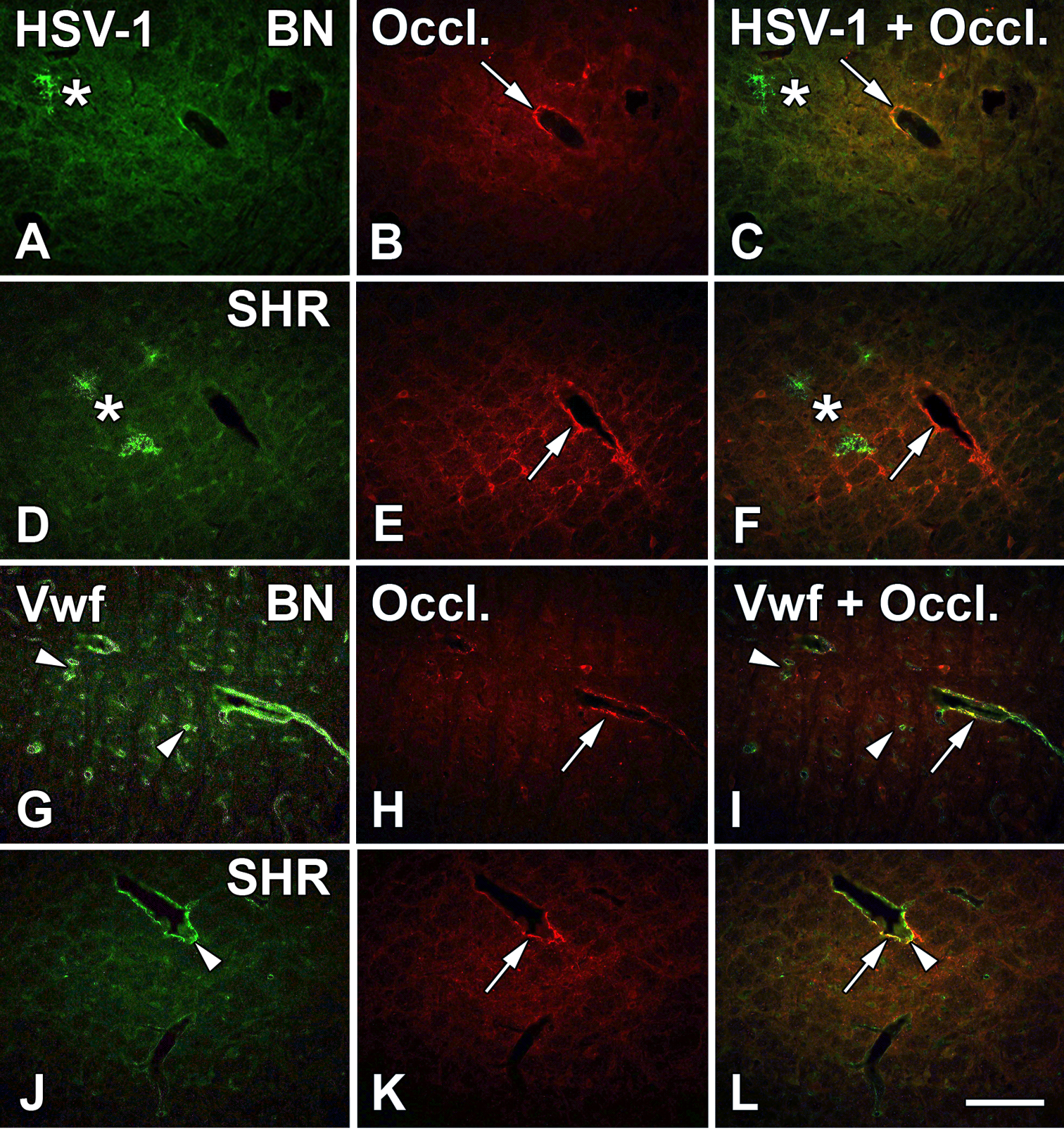

Supplement: S1 Fig — Transversal/coronal sections from the brain stem of BN (A, B, C, G, H and I) and SHR (D, E, F, J, K and L) rats stained with HSV-1 Ab (green) (A, C, D and F), occludin (tight junctions marker) (red) (B, C, E, F, H, I, K and L) and vWF marker (green) (G and J). The HSV-1 spread was seen in the brain stem of the resistant BN rats (A and C; asterisks) and the susceptible SHR (D and F; asterisks). vWF protein was found in the large vessels and capillaries of BN rats in the brain stem (G and I; arrowheads and arrow) while in the SHR rats vWF staining was only visible in the larger vessels (J and L; arrowheads). Occludin staining of the tight junctions was similar in the brain stem of both BN (B, C, H and I; arrows) and SHR (E, F, K and L; arrows) rats. Scale bar: 50 μm. (TIF) [file pone.0155832.s001.tif]
