# Supplementary material for: Von Willebrand Factor Gene Variants Associate with Herpes simplex Encephalitis
Source: PLoS One. 2016 May 25;11(5):e0155832. doi: 10.1371/journal.pone.0155832 (PMC4880288; doi:10.1371/journal.pone.0155832)
Supplement: S1 Table — (DOC) [file pone.0155832.s002.doc]

**Supplementary Table 1. Summary of identified suggestive QTLs that regulate HSE phenotypes in RILs**

| **QTL** | **Chr** | **Level of significance** | **Phenotype** | **Peak Marker** | **Pos**  **(cM)** | **Pos**  **(Mb)** | **LRS** | **LOD** | **Confidence interval (Markers)** | **Position**  **(Mb)** | **Overlapping QTLs** |
| --- | --- | --- | --- | --- | --- | --- | --- | --- | --- | --- | --- |
|  |  |  |  |  |  |  |  |  |  |  |  |
| *Hse7* | 4 | Suggestive | Incidence | D4Rat202 | 69.6 | 159.3 | 12.5 | 2.7 | A2m – Eno2 | 158 – 160.9 | *Pia7* (APLEC locus) |
|  | 4 |  | Wl d0 – d10 | D4Rat202 | 69.6 | 159.3 | 12.4 | 2.7 | A2m – Eno2 | 158 – 160.9 |  |
|  |  |  |  |  |  |  |  |  |  |  |  |
| *Hse8* | 4 | Suggestive | Incidence | D4Rat58 | 57.3 | 136.6 | 11.4 | 2.5 | D4Rat44 – D4Rat240 | 121.8 – 148.2 | *Eae22* (145.7-187Mb) |
|  | 4 |  | Onset | Ampp | 58.9 | 140 | 13.1 | 2.9 | D4Rat44 – D4Rat240 | 121.8 – 148.2 | *Pia9,7 and 23* |
|  | 4 |  | Wl d0 – d10 | D4Rat58 | 57.3 | 136.6 | 12.2 | 2.7 | D4Rat44 – D4Rat240 | 121.8 – 148.2 |  |
|  |  |  |  |  |  |  |  |  |  |  |  |
| *Hse9* | 4 | Suggestive | Incidence | D4Rat176 | 50.7 | 120.5 | 9.2 | 2.0 | Spr – D4rat44 | 119 – 121.9 |  |
|  | 4 | Suggestive | Survival days | D4Rat176 | 50.7 | 120.5 | 9.2 | 2.0 | Spr – D4rat44 | 119 – 121.9 |  |
|  | 4 | Suggestive | Wl d0 – d10 | D4Rat176 | 50.7 | 120.5 | 9.06 | 2.0 | Spr – D4rat44 | 119 – 121.9 |  |
|  |  |  |  |  |  |  |  |  |  |  |  |
| *Hse10* | 1 | Suggestive | Survival days | D1Rat30 | 43.4 | 100.6 | 10.6 | 2.3 | Klk1 – D1Rat268 | 94.4 – 109.3 |  |
|  |  |  |  |  |  |  |  |  |  |  |  |
| *Hse11* | 4 | Suggestive | Incidence | D4Cebr28s5 | 27.1 | 60 | 10.7 | 2.3 | D4Mit9 – D4Cebr215s9 | 54.8 – 64 |  |
|  | 4 | Suggestive | Survival days | D4Cebr28s5 | 27.1 | 60 | 11.0 | 2.4 | D4Mit9 – D4Cebr215s9 | 54.8 – 64 |  |
|  | 4 | Suggestive | Wl d0 – d10 | D4Cebr28s5 | 27.1 | 60 | 10.6 | 2.3 | D4Mit9 – D4Cebr215s9 | 54.8 – 64 |  |
|  |  |  |  |  |  |  |  |  |  |  |  |
| *Hse12* | 4 | Suggestive | Survival days | D4Rat169 | 40 | 81 | 8.7 | 1.9 | Hoxa – D4Rat153 | 80.7 – 90 | *Eae25* (78- 82.2 Mb) |
|  | 4 | Suggestive | Wl d0 – d5 | D4Rat169 | 40 | 81 | 9.6 | 2.1 | Hoxa – D4Rat153 | 80.7 – 90 | *Eae26* (88.4- 104.4 Mb) |
|  | 4 | Suggestive | Wl d4 – d5 | D4Rat169 | 40 | 81 | 11.0 | 2.4 | Hoxa – D4Rat153 | 80.7 – 90 |  |
|  |  |  |  |  |  |  |  |  |  |  |  |
| *Hse13* | 9 | Suggestive | Wl d4– d5 | D9Utr31 | 39.8 | 78 | 8.9 | 1.9 | Inha – Alpi | 74.7 – 85.8 |  |
|  |  |  |  |  |  |  |  |  |  |  |  |
| *Hse14* | 10 | Suggestive | Incidence | D10Mit6 | 4.5 | 14.7 | 9.2 | 2.0 | D10Cebr27s2 – D10Rat121 | 12 – 16.8 |  |
|  | 10 | Suggestive | Survival days | D10Mit6 | 4.5 | 14.7 | 10.6 | 2.3 | D10Cebr27s2 – D10Rat121 | 12 – 16.8 |  |

Values represent LRS (likelihood ratio scores) for main effect QTLs. Significance thresholds were generated with 1000 permutations and 1000 bootstraps. The peak marker denoted to the marker closest to the position that showed the maximum LRS for each trait. To convert LRS to LOD scores, divide by 4.6. Eae- experimental allergic encephalomyelitis; Pia- pristane induced arthritis
