# Supplementary material for: Von Willebrand Factor Gene Variants Associate with Herpes simplex Encephalitis
Source: PLoS One. 2016 May 25;11(5):e0155832. doi: 10.1371/journal.pone.0155832 (PMC4880288; doi:10.1371/journal.pone.0155832)
Supplement: S2 Table — (DOC) [file pone.0155832.s003.doc]

**Supplementary Table 2. List of SNPs within the *cis*-regulated genes in *Hse6*.**
