# Supplementary material for: Von Willebrand Factor Gene Variants Associate with Herpes simplex Encephalitis
Source: PLoS One. 2016 May 25;11(5):e0155832. doi: 10.1371/journal.pone.0155832 (PMC4880288; doi:10.1371/journal.pone.0155832)
Supplement: S3 Table — (DOCX) [file pone.0155832.s004.docx]

**Supplementary Table 3. Summary of VWF SNPs analysis and association in HSE cases and EIMS controls using recessive test.**

|  |  |  | **Scandinavian** | **All individuals** |
| --- | --- | --- | --- | --- |
| **SNP** | **Position** | **Minor allele** | **Recessive Test P-value** | **Recessive Test P-value** |
| RS2270151 | 5931221 | A | NA | NA |
| RS2286646 | 5931752 | G | 0.6328 | 0.5005 |
| RS12317523 | 5933403 | A | 0.9647 | 0.8985 |
| RS723188 | 5934166 | A | 0.6481 | 0.8702 |
| RS12368267 | 5941777 | G | NA | NA |
| RS11063961 | 5944277 | G | 0.8864 | 0.9412 |
| RS12300917 | 5949319 | A | NA | NA |
| RS917857 | 5952085 | A | 0.3238 | 0.381 |
| **RS917859** | 5952374 | A | **0.003122** | **0.001279** |
| RS4764521 | 5954584 | A | 0.8172 | 0.754 |
| RS216883 | 5967918 | A | 0.7541 | 0.6775 |
| RS216889 | 5969714 | A | 0.7723 | 0.9946 |
| RS17491334 | 5974105 | A | NA | NA |
| RS216312 | 5999245 | A | 0.9961 | 0.6849 |
| RS11611917 | 6006895 | A | 0.9041 | 0.8215 |
| RS216338 | 6018587 | A | 0.7224 | 0.5116 |
| RS980130 | 6039288 | A | 0.4368 | 0.4277 |
| RS4764482 | 6039994 | G | 0.6965 | 0.06278 |
| RS12319392 | 6040747 | A | NA | NA |
| RS2283332 | 6044990 | A | NA | NA |
| RS3782711 | 6053442 | G | NA | NA |
| RS2238104 | 6057926 | A | 0.15 | 0.3248 |
| RS2239140 | 6070704 | A | 0.5602 | 0.6188 |
| RS11064024 | 6072310 | G | 0.2814 | 0.1737 |
| RS11836843 | 6073079 | G | NA | NA |
| RS1860545 | 6317038 | A | 0.8265 | 0.7791 |

-Tagging was not performed with the recessive test. NA=not applicable. Success rate for each marker in cases and controls was between (98 – 100%). Chr12 SNPs positions (in bp) (Genome build 36.3). Scandinavian column: represent analysis of P-value made in Scandinavian 115 HSE cases and 428 EIMS controls; All Individuals’ column: represent analysis of P-value made in Scandinavian and non-Scandinavian 119 HSE cases and 508 EIMS controls.
